# Rational Design and Synthesis of New Selective COX-2 Inhibitors with In Vivo PGE2-Lowering Activity by Tethering Benzenesulfonamide and 1,2,3-Triazole Pharmacophores to Some NSAIDs

**DOI:** 10.3390/ph15101165

**Published:** 2022-09-20

**Authors:** Nada H. El-Dershaby, Soad A. El-Hawash, Shaymaa E. Kassab, Hoda G. Daabees, Ahmed E. Abdel Moneim, Mostafa M. M. El-Miligy

**Affiliations:** 1 Pharmaceutical Chemistry Department, Faculty of Pharmacy, Damanhour University, Damanhour 22516, Egypt; 2 Pharmaceutical Chemistry Department, Faculty of Pharmacy, Alexandria University, Alexandria 21521, Egypt; 3 Department of Organic and Medicinal Chemistry, Faculty of Pharmacy, University of Sadat City, Sadat City 32897, Egypt; 4 Department of Zoology and Entomology, Faculty of Science, Helwan University, Cairo 11795, Egypt

**Keywords:** sulfonamides, 1,2,3-triazole, NSAIDs, in vivo anti-inflammatory, COX-2 inhibitors

## Abstract

New selective COX-2 inhibitors were designed and synthesized by tethering 1,2,3-triazole and benzenesulfonamide pharmacophores to some NSAIDs. Compounds **6b** and **6j** showed higher in vitro COX-2 selectivity and inhibitory activity (IC_50_ = 0.04 µM and S.I. = 329 and 312, respectively) than celecoxib (IC_50_ = 0.05 µM and S.I. = 294). Compound **6e** revealed equipotent in vitro COX-2 inhibitory activity to celecoxib. Furthermore, **6b** and **6j** expressed more potent relief of carrageenan-induced paw edema thickness in mice than celecoxib, with ED_50_ values of 11.74 µmol/kg and 13.38 µmol/kg vs. 16.24 µmol/kg, respectively. Compounds **6b** and **6j** inhibited the production of PGE2 with a % inhibition of PGE2 production of 90.70% and 86.34%, respectively, exceeding celecoxib’s percentage (78.62%). Moreover, **6b** and **6j** demonstrated a gastric safety profile comparable to celecoxib. In conclusion, compounds **6b** and **6j** better achieved the target goal as more potent and selective COX-2 inhibitors than celecoxib in vitro and in vivo.

## 1. Introduction

Conventional nonsteroidal anti-inflammatory drugs (NSAIDs) constitute the vast majority of utilized classes of therapeutic drugs. They provide pain relief together with treating inflammatory disorders, including inflammatory joint diseases and rheumatoid arthritis [1]. 

NSAIDs exert their effect by blocking the proinflammatory prostaglandin (PG) biosynthesis from arachidonic acid (AA) due to their potential to inhibit cyclooxygenase (COX) isoforms: the constitutive COX-1 and the inducible COX-2 [2]. COX-1 is an intrinsic enzyme responsible for maintaining gastric and renal homeostasis and mainly disseminated in the stomach, kidney and platelets, whereas COX-2 is an inducible enzyme, expressed as a result of tissue damage or inflammation [3]. 

Hence, nonselective COX inhibition has been associated with common NSAID side effects, mainly gastrointestinal side effects and renal impairment due to COX-1 inhibition. As a result, traditional NSAIDs were unfavorable in cases of chronic inflammation [4]. On the other hand, selective COX-2 inhibitors expressed minor gastrointestinal disorders in comparison with traditional NSAIDs, offering improved safety in chronic cases. However, other side effects manifested, mainly the cardiovascular risks that led to the withdrawal of some coxibs from the pharmaceutical market [5,6,7].

Consequently, several research groups have focused on the discovery of selective scaffolds for COX-2 inhibition with an improved safety profile [8]. It had been reported that the selectivity of COX-2 inhibitors is assigned to the phenyl sulfonamide moiety due to its binding to the polar side pocket available in the COX-2 enzyme but not in COX-1, and which remains unoccupied in complexes of nonselective COX inhibitors with COX-2 [9]. Away from the hydrophobic pocket, the sulfonamide group protracts into the COX-2 polar side (selective) pocket. The sulfonamide links to Gln178, Leu338 and Ser339 amino acid residues of the polar side pocket by forming hydrogen bonds [10]. In contrast, various nonselective COX inhibitors are characterized by the presence of a carboxylate group responsible for salt-bridge formation with the basic nitrogen of Arg120 residue that has been reported as an important factor for COX inhibition [11]. Hence, the deletion of the carboxylic group of some nonselective inhibitors may enhance their COX-2 selectivity as the formation of the salt bridge with the Arg120 residue of COX-1 may be inaccessible. 

**Figure 1:**
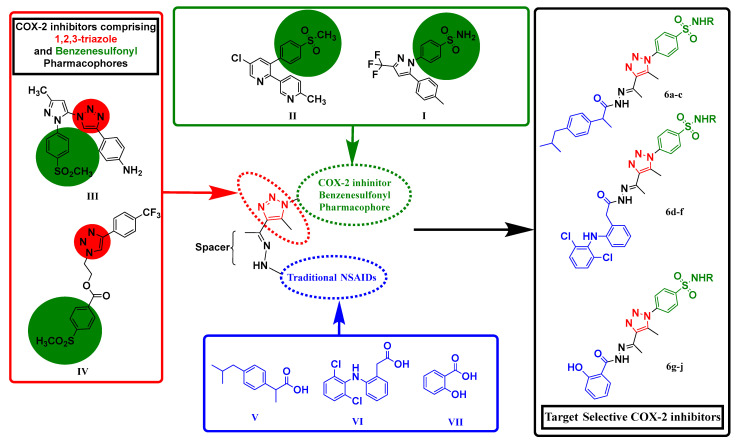
Rational design of the target selective COX-2 inhibitors.

It is worth mentioning that some NSAIDs containing the sulfonamide or sulfone moiety were commercially available with reduced cardiovascular events such as celecoxib (**I**) and etoricoxib (**II**) (Figure 1), whereas rofecoxib was withdrawn from the market [12].

Moreover, the 1,2,3-triazole moiety has gained great attention in medicinal chemistry as a bioisostere of esters, amides and carboxylic acids. Accordingly, it interacts with various proteins, enzymes and receptors in different organisms via weak bond interactions such as hydrogen bonds, hydrophobic interactions and van der Waals forces [13,14].

Moreover, hybridization between 1,2,3-triazole and benzenesulfonyl pharmacophores in compounds **III** and **IV** (Figure 1) have been reported as potent and selective COX-2 inhibitors [15,16]. 

Consequently, the current study aims at the development of new hybrid molecules possessing potent and selective COX-2 inhibitory activity with reduced side effects. In fact, our study is mainly based on blocking the carboxylic group of some traditional NSAIDs—ibuprofen (**V**), diclofenac (**VI**) and salicylic acid (**VII**) (Figure 1)—by converting it to acid hydrazide to decrease the interaction with COX-1 isozyme. Furthermore, the hydrazides were bound to 1,2,3-triazole-benzenesulfonamide hybrids to increase selectivity to COX-2 (Figure 1). 

In addition, the newly synthesized ligands were evaluated for their in vitro COX-1/2 inhibitory activities. The most active compounds were further screened in vivo to assess their anti-inflammatory potential inside the biological system together with their ulcerogenic and PGE2-lowering activity. Moreover, docking studies were conducted to define the binding pattern of the newly synthesized compounds within the active site of the targeted enzyme (COX-2). Furthermore, in silico prediction of physicochemical and pharmacokinetic parameters was performed to evaluate their capacity as drug/lead-like candidates. Finally, ligand efficiency (LE) and lipophilic ligand efficiency (LLE) were estimated to measure the influence of the molecule’s structural features on the efficient binding to the target and to normalize the in vitro biological activity with respect to the physicochemical properties of the molecule.

## 2. Results and Discussion

### 2.1. Chemistry

**Scheme 1:**
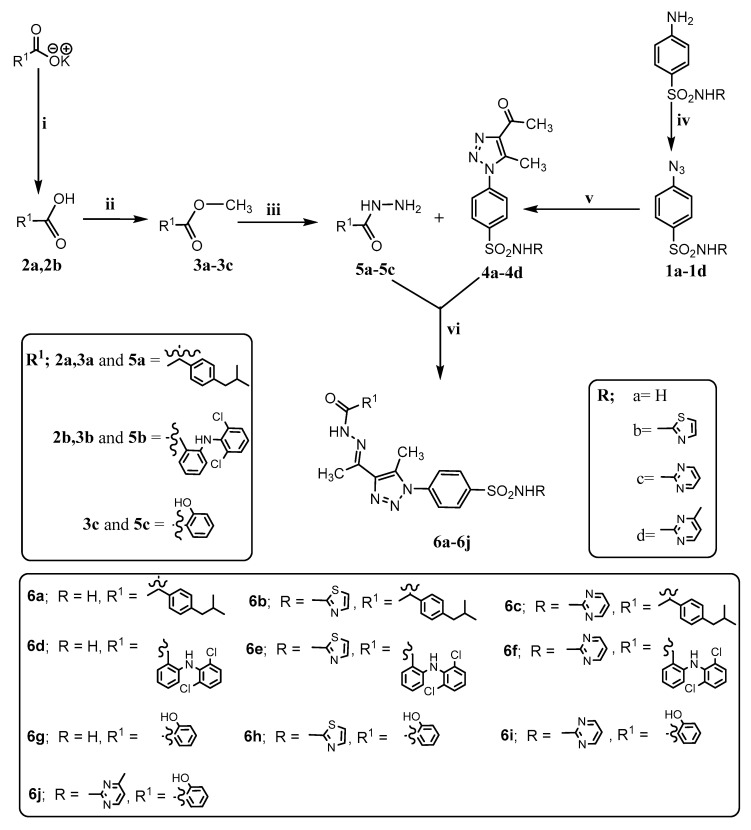
Reagents and conditions: **i**: 2N HCl; **ii**: CH_3_OH, H_2_SO4, reflux, 12 h; **iii**: NH_2_NH_2_.H_2_O 99%, C_2_H_5_OH, reflux, 5 h; **iv**: HCl, NaNO_2_, NaN_3_, 0 °C, stir overnight; **v**: CH_3_COCH_2_COCH_3_, CH_3_ONa, CH_3_OH, stir overnight; **vi**: glacial CH_3_COOH, C_2_H_5_OH, reflux, 8–12 h.

The synthetic routes anticipated to obtain the intermediates as well as the target compounds are outlined in Scheme 1. 

As shown in Scheme 1, diazotization of heteroaryl sulfonamides and subsequent treatment with sodium azide has been employed for synthesis of aryl azides (**1a**–**d**), as reported in [17].

The structure of the new intermediate **1c** was confirmed by the presence of a characteristic D_2_O exchangeable singlet signal at δ 9.06 ppm corresponding to the sulfamoyl NH proton, whereas the intermediate **1d** was characterized by a singlet signal at δ 2.32 ppm corresponding to three protons of pyrimidinyl-4-CH_3_ together with the D_2_O exchangeable singlet of sulfamoyl NH proton at δ 9.30 ppm according to the ^1^H NMR spectra. 

We synthesized 1,4,5-trisubstituted 1,2,3-triazoles via azide–enolate cycloaddition, as previously reported [18]. Reacting aryl azides (**1a**–**d**) with acetylacetone in the presence of sodium methoxide/methanol afforded the key intermediates, 4-acetyl-1,2,3-triazole derivatives (**4a**–**d**), in good yields. The entity of intermediate **4c** was confirmed by the ^1^H NMR spectrum which revealed two singlets at δ 2.44 ppm and 2.58 ppm corresponding to the acetyl and 5-methyl protons, respectively. Additionally, the ^1^H NMR spectrum of the intermediate **4d** was characterized by three singlet signals at δ 2.07 ppm, 2.72 ppm and 2.80 ppm corresponding to acetyl, pyrimidinyl-4-CH_3_ protons and triazolyl-5-CH_3_ protons, respectively, together with one D_2_O exchangeable singlet signal at δ 12.40 ppm corresponding to the sulfamoyl NH proton. The ^13^C NMR spectrum of **4d** revealed signals at δ 9.84 ppm, 21.10 ppm and 23.06 ppm corresponding to the three aliphatic carbons: triazolyl-5-CH_3_, pyrimidinyl-4-CH_3_ and triazolyl-4-COCH_3_, respectively, as well as the characteristic signal at δ 193.38 ppm for the carbonyl group. 

Moreover, diclofenac potassium was neutralized by 2N HCl to produce the corresponding acid (diclofenac) (**2b**) following reported procedures [19]. Both diclofenac and ibuprofen were converted to their methyl esters (**3a,b**) using standard methods for esterification [20]. Furthermore, methyl esters of diclofenac, ibuprofen and salicylic acid (**3a**–**c**) were treated with hydrazine hydrate to produce the corresponding acid hydrazides (**5a**–**c**) according to the reported procedures [21]. 

The target compounds (**6a**–**j**) were attained by condensation of the appropriate methyl ketones (**4a**–**d**) with acid hydrazides (**5a**–**c**) in the presence of glacial acetic acid in ethanol. 

The integrity of the structures of the newly synthesized compounds was confirmed using elementary microanalyses: IR, ^1^ H NMR, ^13^C NMR and mass spectrometry (see the Experimental section and the Supporting Information). 

The IR spectra of compounds **6a**–**j** revealed absorption bands characteristic of OH, NH, C=O, C=N and SO_2_ functional groups. Moreover, the ^1^H-NMR spectra of compounds **6a**–**j** were characterized by the presence of two characteristic D_2_O exchangeable singlet signals at δ 12.04–12.48 ppm and δ 10.64–11.40 ppm corresponding to the amide CO-NH proton and sulfamoyl NH proton, respectively.

The ^1^H-NMR spectra of compounds **6a**–**c** disclosed the presence of signals characteristic of the isobutyl group, including a doublet signal at 0.99 ppm corresponding to the six protons of isobutyl-C_3, 4_ protons, a multiplet signal at δ 1.80–1.88 ppm corresponding to the isobutyl-C_2_proton and a doublet signal at δ2.83 ppm for the two isobutyl-C_1_ protons, in addition to a doublet signal at δ 1.43 ppm corresponding to the three protons of CO-CH-CH_3_, two singlets at δ 2.33 ppm and 2.66 ppm each corresponding to three protons of N=C-CH_3_ and triazolyl-5-CH_3_, respectively, as well as a quartet signal at δ 3.90 ppm corresponding to the CO-CH proton. 

Furthermore, compounds **6d**–**f** were characterized by three singlet signals at δ 2.42 ppm, 2.68 ppm and 3.65 ppm corresponding to the three methyl protons of N=C-CH_3_, the three methyl protons of triazolyl-5-CH_3_ and the two protons of O=C-CH_2_, respectively, in addition to a singlet D_2_O exchangeable signal at δ 9.02–9.42 ppm corresponding to the phenolic NH proton. 

Additionally, compounds **6g**–**j** were confirmed by the presence of a singlet signal at δ 2.58–2.68 ppm corresponding to the three methyl protons of triazolyl-5-CH_3_ as well as the characteristic D_2_O exchangeable singlet signal at δ 11.60–11.81 ppm for the phenolic OH proton. The ^13^C NMR spectra disclosed the presence of a characteristic signal for the carbonyl group at its expected region (δ 167.31–193.39 ppm). Moreover, it revealed the presence of signals at δ 139.28–159.96 ppm corresponding to N=C and signals at δ 9.25–10.44 ppm corresponding to triazolyl-5-CH_3_. Additionally, the presence of signals at δ 22.40 ppm, 30.23 ppm and 44.93 ppm was characteristic of the isobutyl group in compounds **6a**–**c**. Finally, the MS spectra confirmed the molecular ion peak (M^+•^) at *m*/*z* 565.65 for **6b**, at 649.98 for **6f** and at 506.32 for **6j**.

### 2.2. Biological Evaluation

#### 2.2.1. In Vitro Human COX-1 and Human COX-2 Enzymatic Inhibitory Activities

Investigating the potential of the newly synthesized compounds to selectively inhibit human cyclooxygenase-2 isoenzyme (COX-2) was carried out by exploring their in vitro inhibitory activity towards both human COX-1 and human COX-2 isoenzymes. Celecoxib was used as a standard selective human COX-2 inhibitor. In addition, indomethacin and diclofenac sodium were used as standard nonselective human COX inhibitors. The enzymatic assay was carried out according to a procedure in the literature [22,23] utilizing an ovine COX-1/human recombinant COX-2 assay kit (catalog no. 560131; Cayman Chemicals Inc., Ann Arbor, MI, USA). Inhibitory activities were defined as a test compound concentration achieving 50% human COX-1 or human COX-2 inhibition. The selectivity of compounds was referred to as a compound’s selectivity index values calculated as IC_50_ (COX-1)/IC_50_ (COX-2). The experiments were performed in triplicate and the results are listed in Table 1. 

As shown in Table 1, all the compounds displayed potent human COX-2 inhibition compared to indomethacin and diclofenac, with IC_50_ values of (0.04–0.11 µM). Moreover, compounds **6a**–**j** expressed weak human COX-1 enzymatic inhibition in comparison to both indomethacin and diclofenac. Surprisingly, compounds **6b**, **6e** and **6j** were discovered as human COX-2 inhibitors with inhibitory profiles comparable to celecoxib (IC_50_ 0.04 µM for **6b** and **6j** and 0.05 µM for **6e** vs. 0.05 µM for celecoxib). The rest of the compounds exerted about 45–71% of the human COX-inhibitory activity of celecoxib. Interestingly, two compounds, namely **6b** and **6j**, expressed prominent human COX-2 selectivity indices of 329 and 312, respectively, excelling that of celecoxib (SI = 294). Meanwhile, the rest of the compounds showed selectivity indices exceeding those of nonselective human COX-inhibitors, while they were less for selective human COX-2 inhibitors than celecoxib. Thus, they were expected to produce less gastric irritation associated with nonselective human COX-inhibitors. According to the structures of the newly synthesized compounds, condensation of the starting triazolyl ethanone **4d** with salicylic acid hydrazide **5c** into the hydrazone **6j** produced a noticeable improvement in human COX-2 inhibitory activity (IC_50_ value of 0.11 µM vs. 0.04 µM, respectively). Furthermore, hybridization between triazolyl ethanones **4a**–**c** and diclofenac acid hydrazide **5b** which afforded analogues **6d**–**f** proved to be more selective human COX-2 inhibitors than diclofenac sodium (SI in the range of 105–204.6 vs. 4.52, respectively). In addition, the thiazolyl benzenesulfonamide analogue **6b** exhibited more potent human COX-2 inhibition than the sulfonamide analogues **6a** and **6c**. Moreover, the human COX-2 inhibitory activity of the thiazolyl benzenesulfonamide analogue **6e** exceeded that of the **6d** and **6f** analogues.

#### 2.2.2. In Vivo Carrageenan-Induced Paw Edema in Mice

**Figure 2:**
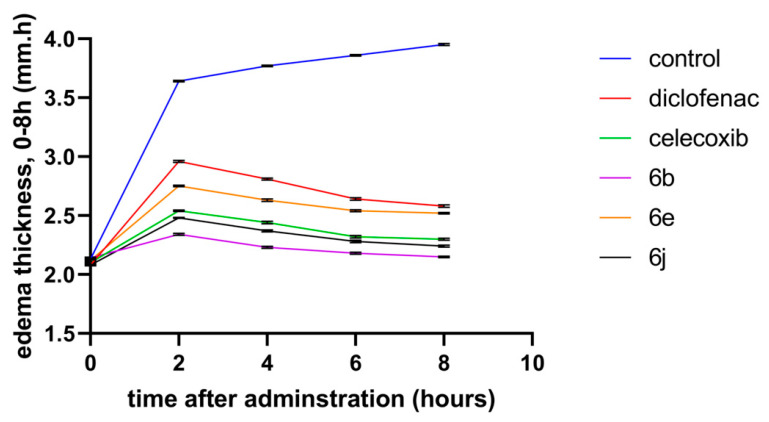
Effects of the tested compounds **6b**, **6e** and **6j** as well as celecoxib and diclofenac as positive controls on the thickness of carrageenan-induced paw edema in mice along interval of 0–8 h after carrageenan injection (mm) in a dose of 10 µmol/kg.

The aforementioned in vitro data revealed that the ibuprofen analogue **6b**, the salicylic acid analogue **6j** together with the diclofenac analogue **6e** expressed excellent human COX-2 selectivity and inhibitory activities. Hence, an in vivo carrageenan-induced paw edema bioassay in mice was implemented [24,25] on the three analogues **6b**, **6j** and **6e** in order to fulfill their anti-inflammatory potency. The capacity of the targeted compounds to relieve mouse paw edema thickness (mm) during specified time intervals (2, 4, 6 and 8 h) after injecting carrageenan was measured, and the protection percent against edema (anti-inflammatory activity; AI%) was calculated. Celecoxib and diclofenac were taken as positive controls (Table 2 and Figure 2). The collected results showed that the ibuprofen analogue **6b** expressed more rapid onset of action than both celecoxib and diclofenac, revealed from its effect after 2 h exceling that of celecoxib and diclofenac. Moreover, the salicylic acid derivative **6j** expressed a similar pharmacokinetic profile to that of celecoxib, manifested in their similar % of edema inhibition after 2 h of carrageenan injection (73.51% vs. 70.86%). Considering their effects after 8 h as significant criteria for comparison, it can be said that hybridizing the anti-inflammatory drug, ibuprofen, with 4-acetyl-1,2,3-triazole derivative (**4b**) succeeded in improving the anti-inflammatory activity of the resulting analogue **6b**. It completely got rid of carrageenan-induced edema with the maximum % of edema inhibition among the series. Surprisingly, the three evaluated compounds **6b**, **6e** and **6j** showed more potent inflammatory inhibition than the nonselective COX inhibitor, diclofenac. To conclude, compounds **6b** and **6j** displayed the maximum percentage of anti-inflammatory activity, exceeding both celecoxib and diclofenac. Compounds **6b**, **6e** and **6j** were further screened at 5, 10, 20, 30 and 40 µmol/kg in order to plot dose–response curves and calculate their ED_50_ values, the median effective dose (Table 2). According to data in Table 2, two compounds, namely **6b** and **6j**, expressed the highest anti-inflammatory activity, exceeding the activity of celecoxib. In fact, the ibuprofen analogue **6b** was found to be the most potent among the tested compounds. Furthermore, the diclofenac analogue **6e** showed a similar ED_50_ value to that of diclofenac (18.23 vs. 18.35 µmol/kg, respectively). 

#### 2.2.3. In Vivo Estimation of Rat Serum Prostaglandin E2 (PGE2)

**Figure 3:**
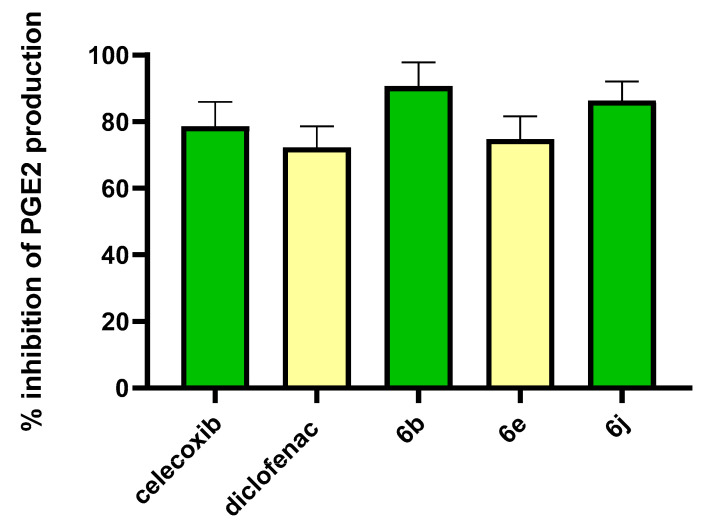
The % inhibition of rat serum prostaglandin E2 production after 8 h of injecting 10 µmol/kg b.wt of the tested compounds as well as celecoxib and diclofenac as positive controls.

Measuring plasma levels of prostaglandin E2 is one of the substantial parameters in confirming the in vivo COX-2 inhibition. Hence, the serum levels of prostaglandin E2 of carrageenan-injected rats treated with the COX-2 inhibitors **6b**, **6e** and **6j** were measured utilizing celecoxib and diclofenac as standard inhibitors (Table 3 and Figure 3). The results showed maximum anti-inflammatory potency with the ibuprofen analogue **6b** and the salicylic acid derivative **6j**. Consequently, they were the most potent PGE2 inhibitors in the series superior to celecoxib. Moreover, the diclofenac derivative **6e** expressed similar potency as a PGE2-lowering agent to the potency of diclofenac.

#### 2.2.4. Ulcerogenic Effects

**Figure 4:**
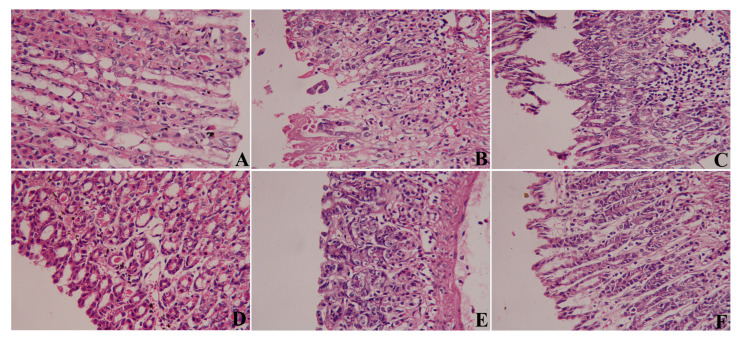
Hematoxylin and eosin (H&E) of stomach sections after the treatment with tested compounds and positive controls. (**A**) The stomach of control rats; (**B**) the stomach of rats treated with celecoxib showing normal gastric texture; (**C**) stomach of rats treated with diclofenac showed severe gastric damage where the gastric mucosa showed thinning, paleness, erosion and irregular arrangement; (**D**) stomach of rats treated with **6b** showed normal gastric texture; (**E**) stomach of rats treated with **6e** showed severe pathogenic changes; (**F**) stomach of rats treated with **6j** showed normal gastric texture.

Careful inspection of the stomach sections isolated from fasted rats after treatment with the tested compounds, as well as celecoxib and diclofenac as reference drugs (Figure 4), disclosed the safety gastric profiles of compounds **6b** and **6j** as well as the selective COX-2 inhibitor, celecoxib. Moreover, the reference drug diclofenac expressed severe gastric injury associated with diffuse inflammatory cells mainly in the mucosal and submucosal layers. Similarly, rat stomach treated with compound **6e** showed signs of gastric inflammatory reactions. 

### 2.3. Docking Studies of the Potential Selective COX-2 Target Inhibitors vs. the Weaker Selective Inhibitors

We docked the target triazoles (**6b**) and (**6j**), which showed potential COX-2 inhibiting activity in the in vitro testing (Table 1), into the COX-2 active site (pdb entry 3LN1) [26] using Molecular Operating Environment (MOE) version 2016.0802. Moreover, we docked the triazole derivative (**6i**) which is categorized as a weaker selective COX-2 inhibitor according to the in vitro testing data results (Table 1).

**Figure 5:**
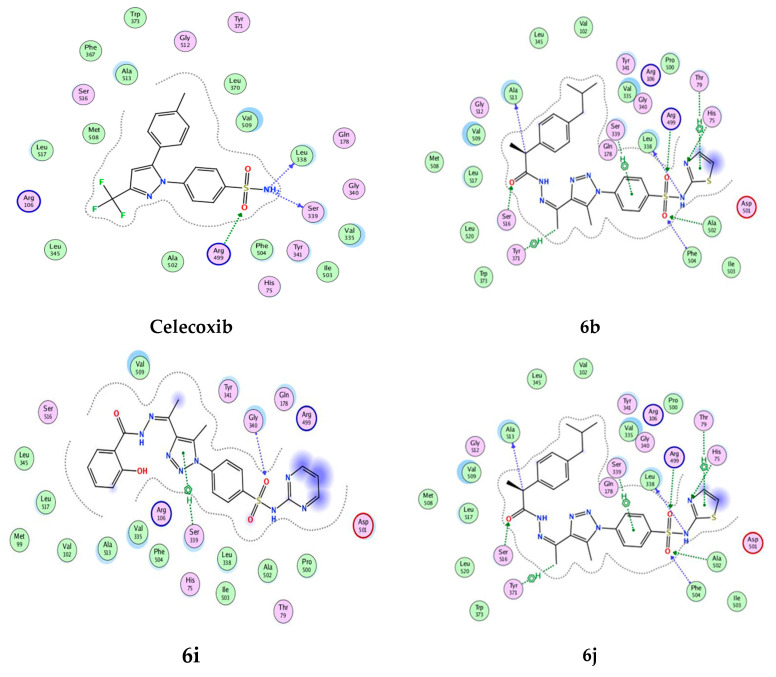

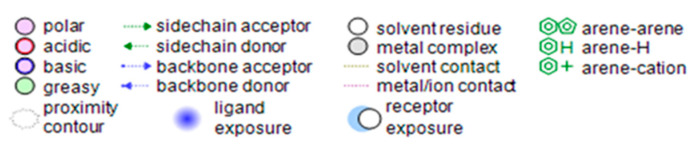
Two-dimensional style of docking solutions of the potential selective COX-2 target inhibitors, **6b** and **6j**; and **celecoxib** as the cocrystallized ligand inhibitor in the active site of COX-2 (PBD code 3LN1). Lastly, the docking solution of compound (**6i**) as a weaker selective COX-2 inhibitor to visualize its binding pattern with the same active site. Amino acid residues represented by the protein sequence code of three letters and numbers; interaction forces represented by green dotted lines which are categorized according to the description scheme associated with the figure.

The docking solutions of the compounds (**6b**) and (**6j**) (Figure 5) verified the potential activities against COX-2 because the drug–receptor interactions of the best poses are very comparable to that of the cocrystallized ligand inhibitor, celecoxib (Figure 5). Both the docked compounds interacted via the sulfamoyl group with the Leu338 and Arg499 amino acids and via the triazole ring with the Ser339 amino acid, which have been previously reported [9] as a part of the polar side pocket of the COX-2 active site. Interaction with this small pocket’s amino acids is essential for the selective inhibition of the enzyme [27]. On the other side, we docked compound (**6i**) as one of the compounds of a weaker selective COX-2-inhibiting activity (Table 1) to understand the reasons behind such lower selectivity. The docking solution of (**6i**) showed a failure of the compound to relax freely into the active site in order to interact with the polar side pocket amino acids, except for Ser339 via alky-π stacking. Interestingly, the presence of the phenolic-OH group might have enabled the triazole derivative (**6i**) to interact with Arg120 as an alternative path to inhibit the enzyme [9].

### 2.4. In Silico Prediction of the Physicochemical Properties, Drug-Likeness Score, Pharmacokinetics, Toxicity Profile and Ligand Efficiency Metrics

One of the vital parameters that can save effort, time and cost as well as facilitate the drug development process is the early prediction of the pharmacokinetic profile and physicochemical properties of new drug candidates through which the lead optimization step can be dramatically enhanced [28]. Herein, the term “drug-likeness” explains the integrated balance of the compound’s structural features with its variable molecular characteristics, so as to be comparable with known drugs. Lipinski’s rule of five (RO5) [29] and Veber’s criteria [30] are among the prevalent principles used to assess drug-like characteristics, including the molecular size, hydrophobicity, flexibility (number of rotatable bonds; nROTB), H-bonding capacity and electronic distribution. Therefore, in silico physicochemical properties, drug-likeness score, pharmacokinetic profile and toxicity, in addition to ligand efficiency metrics of the most active and safest compounds **6b** and **6j**, were investigated using web-based applications, including Molinspiration [31], Pre-ADMET [32] and Osiris property explorer [33] software (Table 4). The results, listed in Table 4, showed acceptable oral bioavailability of compound **6b** that obeys Lipinski’s rule, whereas compound **6j** violated Lipinski’s rule. Moreover, the tested compounds complied with Veber’s criteria, showing nROTB values of 7 and 10 (≤10) and a topographical polar surface area (TPSA; a sum of polar atoms’ surfaces: a descriptor for drug absorption, penetrability and bioavailability), except for **6j**, which exceeded the acceptable limit for TPSA (164.36 Å2) (≤140 Å2). Additionally, the percentage of absorption (% ABS; calculated as % ABS = 109 − 0.345 × TPSA) [34] was calculated showing a considerable oral absorption of the two compounds with % ABS values of 63.72% and 52.30%, respectively. Furthermore, their solubility values were about 0.011 and 4.81 mg/L, respectively, complying with the solubility requirement (>0.0001 mg/L) [35,36], which indicated their good oral absorption and transport characteristics. Concerning drug-likeness score values [37], the evaluated compounds displayed positive values of 0.16 and 0.25, respectively. In another context, compound **6b** displayed moderate cell permeability in a Caco-2 cell model (11.54 nm/s) as compared with the acceptable range (4–70 nm/s), whereas compound **6j** showed low cell permeability in the Caco-2 cell model (0.56 nm/s). Both of them showed low permeability in a MDCK cell model (0.052 and 2.70 nm/s, respectively) compared to the recommended range (25–500 nm/s). Regarding the HIA, the evaluated compounds were well-absorbed, showing high HIA values (91.53 and 96.59%, respectively). Meanwhile, a low BBB permeability coefficient (reported 0.1–2) was displayed by the two compounds, with values of 0.125 and 0.049, respectively. Furthermore, the tested compounds exhibited strong binding to plasma proteins (>90%), showing values of 87.67% and 97.87%, respectively [18]. Other measured metrics related the molecule structural features and physicochemical properties to its potency, including ligand efficiency (LE) and lipophilic ligand efficiency (LLE) [38]. LE values of the two compounds regarding the COX-2 enzyme were 0.26 and 0.28, respectively, which was concordant with the approved minimum LE for lead compounds (around 0.3) [39,40]. LLE values of compound **6j** regarding the COX-2 enzyme (4.87) complied with the acceptable LEE value (≥3) for a lead compound, while compound **6b** displayed a lower LLE value of 2.73. Finally, the predicted toxicity risks revealed that none of the tested compounds showed any irritant effect, mutagenic probability or reproductive toxicity.

## 3. Materials and Methods

### 3.1. Chemistry 

All reagents and solvents were purchased from Sigma-Aldrich, Burlington, MA, United States, and AcrosOrganics-Thermo Fisher Scientific company, Hampton, New Hampshire, Rockingham County, United States, except for ibuprofen and diclofenac potassium which were supplied by Alexandria Co. for pharmaceutical & chemical industries, Alexandria, Egypt. 

The reactions were monitored by thin-layer chromatography (TLC) on silica gel precoated Merck aluminum GF254 plates using Hexane: Ethyl acetate (EtOAc) 7:3 and DCM: MeOH 9.5:0.5 as the eluting systems, and the spots were visualized using Spectroline E series dual wavelength UV lamp at λ = 254 nm. Melting points were measured using open-glass capillaries with a Digital Stuart SMP10 melting point apparatus and are uncorrected. 

Infrared spectra (IR) were recorded on a Thermo Scientific Nicolet iS20 Fourier Transform Infrared Spectrometer using KBr discs, Faculty of Pharmacy, Mansoura University. 

Proton nuclear magnetic resonance spectra (^1^H-NMR) were scanned on a JNM-ECA II 500 MHz JEOL Spectrometer at the NMR unit, Faculty of Science, Mansoura University and on a Bruker Avance III 400 MHz Spectrometer at the Center for Drug Discovery Research and Development, Faculty of Pharmacy, Ein Shams University using deuterated dimethylsulfoxide (DMSO-d_6_) as a solvent. Data were interpreted as chemical shift δ values expressed in parts per million (ppm) relative to tetramethylsilane (TMS) as the internal standard. Signal splitting type was indicated by one of the following letters: s = singlet, d = doublet, t = triplet, q = quartet, dd = doublet of doublet and m = multiplet.

^13^C-NMR spectra were scanned on a JNM-ECA II 125 MHz JEOL Spectrometer at the NMR unit, Faculty of Science, Mansoura University. 

Electron impact mass spectra (EI-MS) were recorded on a Direct Inlet part mass analyzer in Thermo Scientific GCMS model ISQ at the Regional Center for Mycology and Biotechnology, Al-Azhar University, Cairo. Elemental analyses (C, H, N and S) were recorded using FLASH 2000 CHNS/O analyzer, Thermo Scientific, at the Regional Center for Mycology and Biotechnology (RCMB), Al-Azhar University. The results were within ±0.4% of the calculated values for the proposed formulae. 

The new compounds were named according to the naming algorithm developed by CambridgeSoft Corporation using ChemDraw Professional 16.0.1.4. 

Compounds **2b** [19], **3a**–**b** [20], **4a**–**b** [18] and **5a**–**c** [21] were prepared according to the reported procedures**.**

#### 3.1.1. General Procedure for Synthesis of 4-Substituted Phenyl azides (**1a**–**d**)

The appropriate sulfonamide derivative (13 mmol) was suspended in 2N hydrochloric acid (80 mL) and then ethanol was added until a clear solution was obtained. The solution was cooled to 0 °C and NaNO_2_ (1.34 g, 19.5 mmol) was added portionwise. After stirring at 0 °C for 15–30 min, NaN_3_ (1.27 g, 19.5 mmol) was added slowly, and the mixture was stirred overnight at room temperature. The formed precipitate was collected by filtration, washed with aqueous ethanol (1:1), dried and used without further purification.

**4-Azido-N-(pyrimidin-2-yl)benzenesulfonamide (1c)**

Off-white powder; yield: 86%, m.p.: 188–190 °C. IR (KBr, cm^−1^): 3394 (NH), 2105 (N=N=N), 1638 (C=N), 1291, 1139 (SO_2_). ^1^H-NMR (500 MHz, DMSO-d_6_) δ 7.06 (t, *J* = 5.0 Hz, 1H, pyrimidinyl-C_5_-H), 7.41 (d, *J* = 7.9 Hz, 2H, sulfamoyl phenyl-C_3,5_-H), 7.58 (d, *J* = 7.9 Hz, 2H, sulfamoyl phenyl-C_2,6_-H), 8.58 (d, *J* = 5.0 Hz, 2H, pyrimidinyl-C_4,6_-H), 9.06 (s, 1H, sulfamoyl-NH, D_2_O exchangeable). ^13^C-NMR (125 MHz, DMSO-d_6_) δ 114.74 (pyrimidinyl-C_5_), 117.83 (sulfamoyl phenyl-C_3,5_), 129.29 (sulfamoyl phenyl-C_2,6_), 138.16 (sulfamoyl phenyl-C_4_), 142.66 (sulfamoyl phenyl-C_1_), 156.41(pyrimidinyl-C_4,6_), 157.23 (pyrimidinyl-C_2_). Anal.Calcd for C_10_H_8_N_6_O_2_S (276.27): C, 43.47; H, 2.92; N, 30.42; O, 11.58; S, 11.60. Found: C, 43.75; H, 3.02; N, 30.31; O, 11.62; S, 11.68.

**4-Azido-N-(4-methylpyrimidin-2-yl)benzenesulfonamide (1d)**

Pale yellow powder; yield: 80%, m.p.: 197–199 °C. IR (KBr, cm^−1^): 3386 (NH), 2112 (N=N=N), 1643 (C=N), 1319, 1133 (SO_2_). ^1^H-NMR (500 MHz, DMSO-d_6_) δ 2.32 (s, 3H, pyrimidinyl-4-CH_3_), 7.16 (d, *J* = 5.0 Hz, 1H, pyrimidinyl-C_5_-H), 7.42 (d, *J* = 7.9 Hz, 2H, sulfamoyl phenyl-C_3,5_-H), 7.58 (d, *J* = 7.9 Hz, 2H, sulfamoyl phenyl-C_2,6_-H), 8.47 (d, *J* = 5.0 Hz, 1H, pyrimidinyl-C_6_-H), 9.30 (s,1H, sulfamoyl-NH, D_2_O exchangeable). Anal.Calcd for C_11_H_10_N_6_O_2_S (290.30): C, 45.51; H, 3.47; N, 28.95; O, 11.02; S, 11.04. Found: C, 45.62; H, 3.60; N, 28.83; O, 11.12; S, 11.09.

#### 3.1.2. General Procedure for Synthesis of 1-(1-(4-Substituted phenyl)-5-methyl-1H-1,2,3-triazol-4-yl) ethan-1-ones (**4a**–**d**)

To a mixture of the appropriate phenyl azide (**1a**–**d**) (5 mmol) and acetylacetone (10 mmol, 1.02 mL), a solution of sodium methoxide (10 mmol, prepared from 0.23 g of sodium and 13.3 mL of methanol) was added. The reaction mixture was stirred overnight at room temperature and then poured onto crushed ice and neutralized with glacial acetic acid until a precipitate was formed. The precipitate was filtered off, washed with water and crystallized from glacial acetic acid.

**4-(4-Acetyl-5-methyl-1H-1,2,3-triazol-1-yl)-N-(pyrimidin-2-yl)benzenesulfonamide (4c)**

Shiny off-white needles; yield: 73%, m.p.: 233–235 °C, IR (KBr, cm^−1^): 3443 (NH), 1684 (C=O), 1311, 1148 (SO_2_). ^1^H-NMR (500 MHz, DMSO-d6) δ 2.44, (s, 3H, COCH_3_), 2.58 (s, 3H, triazolyl-5-CH_3_), 7.03 (t, *J* = 5.0 Hz, 1H, pyrimidinyl-C_5_-H), 7.83 (d, *J* = 8.5 Hz, 2H, sulfamoylphenyl-C_3,5_-H), 8.16 (d, *J* = 8.5 Hz, 2H,sulfamoylphenyl-C_2,6_-H), 8.49 (d, *J* = 5.0 Hz, 2H, pyrimidinyl-C_4,6_-H), 12.31 (s, 1H, NH, D_2_O exchangeable). Anal.Calcd for C_15_H_14_N_6_O_3_S (358.38): C, 50.27; H, 3.94; N, 23.45; S, 8.95. Found: C, 50.38; H, 4.05; N, 23.33; S, 9.05.

**4-(4-Acetyl-5-methyl-1H-1,2,3-triazol-1-yl)-N-(4-methylpyrimidin-2-yl)benzenesulfonamide (4d)**

Shiny yellow crystals; yield: 70%, m.p.: 241–243 °C, IR (KBr, cm^−1^): 3398 (NH), 1679 (C=O), 1337, 1150 (SO_2_). ^1^H-NMR (500 MHz, DMSO-d6) δ 2.07 (s, 3H, COCH_3_), 2.72 (s, 3H, pyrimidinyl-4-CH_3_), 2.80 (s, 3H, triazolyl-5-CH_3_), 7.09 (d, *J* = 5.0 Hz, 1H, pyrimidinyl-C_5_-H), 8.03 (d, *J* = 8.5 Hz, 2H, sulfamoylphenyl-C_3,5_-H), 8.38 (d, *J* = 8.5 Hz, 2H, sulfamoylphenyl-C_2,6_-H), 8.51 (d, *J* = 5.0 Hz, 1H, pyrimidinyl-C_6_-H), 12.40 (s, 1H, sulfamoyl NH, D_2_O exchangeable). ^13^C-NMR (125 MHz, DMSO-d_6_) δ 9.84 (triazolyl-5-CH_3_), 21.10 (pyrimidinyl-4-CH_3_), 23.06 (COCH_3_), 27.75 (pyrimidinyl-C_5_), 114.35 (sulfamoylphenyl-C_3,5_), 125.50 (sulfamoylphenyl-C_2,6_), 129.25 (sulfamoylphenyl-C_4_), 137.82 (sulfamoylphenyl-C_1_), 138.03 (triazolyl-C_5_), 142.37 (triazolyl-C_4_), 143.05 (pyrimidinyl-C_4_), 156.17 (pyrimidinyl-C_2_), 172.09 (pyrimidinyl-C_6_), 193.38 (C=O). Anal.Calcd for C_16_H_16_N_6_O_3_S (372.40): C, 51.60; H, 4.33; N, 22.57; S, 8.61. Found: C, 51.72; H, 4.44; N, 22.49; S, 8.69.

#### 3.1.3. General Procedure for Synthesis of Compounds **6a**–**j**

To a mixture of acid hydrazide **5a**–**c** (1.5 mmol) in absolute ethanol (20 mL), the appropriate methyl ketone **4a**–**d** (1 mmol) was added. The reaction mixture was acidified with 5 drops of glacial acetic acid, refluxed for 12 h and left to cool to room temperature. The separated solid was filtered off, dried and crystallized from DMF and water.

**4-(4-(1-(2-(2-(4-Isobutylphenyl)propanoyl)hydrazineylidene)ethyl)-5-methyl-1H-1,2,3-triazol-1-yl)benzenesulfonamide (6a)**

Off-white needles; yield: 63%, m.p.: 205–207 °C. IR (KBr, cm^−1^): 3419, 3354 (NH_2_), 3264 (NH), 1671 (C=O), 1523 (C=N), 1343, 1161 (SO_2_). ^1^H-NMR (400 MHz, DMSO-d_6_) δ 0.99 (d, *J* = 6.7 Hz, 6H, two CH_3_ of isobutyl), 1.43 (d, *J* = 6.8 Hz, 3H, CO-CH-CH_3_), 1.80–1.88 (m, 1H, isobutyl-C_2_-H), 2.33 (s, 3H, N=C-CH_3_), 2.66 (s, 3H, triazolyl-5-CH_3_), 2.83 (d, *J* = 6.7Hz, 2H, isobutyl-C_1_-H), 3.90 (q, *J* = 6.8 Hz, 1H, CO-CH), 4.28 (s, 2H, sulfamoyl NH_2_, D_2_O exchangeable), 7.03 (d, *J* = 7.5 Hz, 2H, phenyl-C_3,5_-H), 7.38 (d, *J* = 7.5 Hz, 2H, phenyl-C_2,6_-H), 7.59 (d, *J* = 8.0 Hz, 2H, sulfamoylphenyl-C_3,5_-H), 7.83 (d, *J* = 8.0 Hz, 2H, sulfamoylphenyl-C_2,6_-H), 11.25 (s, 1H, CONH, D_2_O exchangeable). ^13^C-NMR (125 MHz, DMSO-d_6_) δ 9.73 (triazolyl-5-CH_3_), 13.32 (N=C-CH_3_), 18.12 (CO-CH-CH_3_), 22.40 (isobutyl-C_3, 4_), 30.23 (isobutyl-C_2_), 43.74 (CO-CH), 44.93 (isobutyl-C_1_), 121.73 (sulfamoylphenyl-C_3,5_), 127.08 (phenyl-C_2,6_), 128.10 (phenyl-C_3,5_), 129.01 (sulfamoylphenyl-C_2,6_), 134.69 (triazolyl-C_5_), 135.10 (sulfamoylphenyl-C_4_), 135.52 (sulfamoylphenyl-C_1_), 137.75 (phenyl-C_4_), 139.98 (N=C), 141.21 (phenyl-C_1_), 142.09 (triazolyl-C_4_), 170.37 (C=O). Anal.Calcd for C_24_H_30_N_6_O_3_S (482.60): C, 59.73; H, 6.27; N, 17.41; S, 6.64. Found: C, 59.83; H, 6.35; N, 17.33; S, 6.60.

**4-(4-(1-(2-(2-(4-Isobutylphenyl)propanoyl)hydrazineylidene)ethyl)-5-methyl-1H-1,2,3-triazol-1-yl)-N-(thiazol-2-yl)benzenesulfonamide (6b)**

Pale yellow needles; yield: 63%, m.p.: 213–215 °C. IR (KBr, cm^−1^): 3394, 3341 (NH_2_), 3262 (NH), 1639 (C=O), 1565 (C=N), 1331, 1123 (SO_2_). ^1^H-NMR (400 MHz, DMSO-d_6_) δ 0.96 (d, *J* = 6.7 Hz, 6H, two CH_3_ of isobutyl), 1.44 (d, *J* = 6.8 Hz, 3H, CO-CH-CH_3_), 1.80–1.88 (m, 1H, isobutyl-C_2_-H), 2.32 (s, 3H, N=C-CH_3_), 2.65 (s, 3H, triazolyl-5-CH_3_), 2.83 (d, *J* = 6.7 Hz, 2H, isobutyl-C_1_-H), 3.89 (q, *J* = 6.8 Hz, 1H, CO-CH), 6.83 (d, *J* = 4.9 Hz, 1H, thiazolyl-C_5_-H), 7.05 (d, *J* = 7.5 Hz, 2H, phenyl-C_3,5_-H), 7.17 (d, *J* = 7.5 Hz, 2H, phenyl-C_2,6_-H), 7.25 (d, *J* = 4.9 Hz, 1H, thiazolyl-C_4_-H), 7.54 (d, *J* = 8.0 Hz, 2H, sulfamoylphenyl-C_3,5_-H), 7.87 (d, *J* = 8.0 Hz, 2H, sulfamoylphenyl-C_2,6_-H), 11.40 (s, 1H, CONH, D_2_O exchangeable) 12.33 (s, 1H, sulfamoyl NH, D_2_O exchangeable). ^13^C-NMR (125 MHz, DMSO-d_6_) δ 10.28(triazolyl-5-CH_3_), 14.05(N=C-CH_3_), 17.95(CO-CH-CH_3_), 22.23(isobutyl-C_3, 4_), 30.07(isobutyl-C_2_), 43.58(CO-CH), 44.76(isobutyl-C_1_), 108.45 (thiazolyl-C_5_), 120.67(sulfamoylphenyl-C_3,5_), 125.39 (thiazolyl-C_4_), 126.91(phenyl-C_2,6_), 127.94 (phenyl-C_3,5_), 129.07 (sulfamoylphenyl-C_2,6_), 134.53 (triazolyl-C_5_), 136.71 (sulfamoylphenyl-C_4_), 136.76 (sulfamoylphenyl-C_1_), 137.59 (phenyl-C_4_), 139.82 (N=C), 141.04 (phenyl-C_1_), 142.92 (triazolyl-C_4_), 169.04 (thiazolyl-C_2_), 170.20(C=O). Anal.Calcd for C_27_H_31_N_7_O_3_S_2_ (565.71): C, 57.33; H, 5.52; N, 17.33; S, 11.33. Found: C, 57.44; H, 5.62; N, 17.37; S, 11.23. EIMS *m*/*z* (% relative abundance): 566.47 (38.07) (M^+•^ + 1), 565.65 (94.34) (M^+•^), 529.53 (95.71), 499.07 (65.78), 446.37 (67.19), 300.57 (65.06), 255.55 (100) (base peak), 163.49 (73.04), 102.16 (82.59).

**4-(4-(1-(2-(2-(4-Isobutylphenyl)propanoyl)hydrazineylidene)ethyl)-5-methyl-1H-1,2,3-triazol-1-yl)-N-(pyrimidin-2-yl)benzenesulfonamide (6c)**

White crystals; yield: 53%, m.p.: 257–259 °C. IR (KBr, cm^−1^): 3225, 3187 (NH), 1636 (C=O), 1540 (C=N), 1396, 1153 (SO_2_). ^1^H-NMR (400 MHz, DMSO-d_6_) δ 0.97 (d, *J* = 6.7 Hz, 6H, two CH_3_ of isobutyl), 1.44 (d, *J* = 6.8 Hz, 3H, CO-CH-CH_3_), 1.80–1.88 (m, 1H, isobutyl-C_2_-H), 2.33 (s, 3H, N=C-CH_3_), 2.65 (s, 3H, triazolyl-5-CH_3_), 2.85 (d, 2H, *J* = 6.7 Hz, isobutyl-C_1_-H) 3.89 (q, *J* = 6.8 Hz, 1H, CO-CH), 7.03–7.07 (m, 3H, phenyl-C_3,5_-H and pyrimidinyl-C_5_-H), 7.33 (d, *J* = 7.4 Hz, 2H, phenyl-C_2,6_-H), 7.64 (d, *J* = 7.9 Hz, 2H, sulfamoylphenyl-C_3,5_-H), 7.90 (d, *J* = 7.9 Hz, 2H, sulfamoylphenyl-C_2,6_-H), 8.59 (d, *J* = 4.9 Hz, 2H, pyrimidinyl-C_4,6_-H), 11.18 (s, 1H, CONH, D_2_O exchangeable), 12.29 (s, 1H, sulfamoyl NH, D_2_O exchangeable). Anal.Calcd for C_28_H_32_N_8_O_3_S (560.68): C, 59.98; H, 5.75; N, 19.99; S, 5.72. Found: C, 60.06; H, 5.86; N, 19.88; S, 5.62.

**4-(4-(1-(2-(2-(2-((2,6-Dichlorophenyl)amino)phenyl)acetyl)hydrazineylidene)ethyl)-5-methyl-1H-1,2,3-triazol-1-yl)benzenesulfonamide (6d)**

White crystals; yield: 53%, m.p.: 241–243 °C. IR (KBr, cm^−1^): 3344, 3300 (NH_2_), 3235, 3211 (NH), 1625 (C=O), 1516 (C=N), 1417, 1143 (SO_2_). ^1^H-NMR (400 MHz, DMSO-d_6_) δ 2.42 (s, 3H, N=C-CH_3_), 2.68 (s, 3H, triazolyl-5-CH_3_), 3.65 (s, 2H, O=C-CH_2_), 4.47 (s, 2H, sulfamoyl NH_2_, D_2_O exchangeable), 6.36 (dd, *J* = 7.5, 1.9 Hz, 1H, substituted aniline-C_6_-H), 6.75 (ddd, *J* = 7.5, 1.9, 1.4 Hz, 1H, substituted aniline-C_4_-H), 6.91 (ddd, *J* = 7.5, 1.9, 1.4 Hz, 1H, substituted aniline-C_5_-H), 7.06 (t, *J* = 7.5 Hz, 1H, dichloroaniline-C_4_-H), 7.34 (d, *J* = 7.5 Hz, 1H, substituted aniline-C_3_-H), 7.44 (d, *J* = 7.5 Hz, 2H, dichloroaniline-C_3,5_-H), 7.61 (d, *J* = 8.1 Hz, 2H, sulfamoylphenyl-C_3,5_-H), 7.76 (d, *J* = 8.1 Hz, 2H, sulfamoylphenyl-C_2,6_-H), 9.02 (s, 1H, ph-NH, D_2_O exchangeable), 10.91 (s, 1H, CONH, D_2_O exchangeable). Anal.Calcd for C_25_H_23_Cl_2_N_7_O_3_S (572.47): C, 52.45; H, 4.05; N, 17.13; S, 5.60. Found: C, 52.55; H, 4.17; N, 17.04; S, 5.46.

**4-(4-(1-(2-(2-(2-((2,6-Dichlorophenyl)amino)phenyl)acetyl)hydrazineylidene)ethyl)-5-methyl-1H-1,2,3-triazol-1-yl)-N-(thiazol-2-yl)benzenesulfonamide (6e)**

Off-white crystals; yield: 53%, m.p.: 267–269 °C. IR (KBr, cm^−1^): 3356, 3232 and 3162 (NH), 1644 (C=O), 1543 (C=N), 1345, 1149 (SO_2_). ^1^H-NMR (400 MHz, DMSO-d_6_) δ 2.40 (s, 3H, N=C-CH_3_), 2.64 (s, 3H, triazolyl-5-CH_3_), 3.51 (s, 2H, O=C-CH_2_), 6.23 (dd, *J* = 7.5, 1.9 Hz, 1H, substituted aniline-C_6_-H), 6.74 (ddd, *J* = 7.5, 1.9, 1.4 Hz, 1H, substituted aniline-C_4_-H), 6.83 (d, *J* = 5.0 Hz, 1H, thiazolyl-C_5_-H), 6.91 (ddd, *J* = 7.5, 1.9, 1.4 Hz, 1H, substituted aniline-C_5_-H), 7.04–7.08 (m, 2H, substituted aniline-C_3_-H and dichloroaniline-C_4_-H), 7.25 (d, *J* = 5.0 Hz, 1H, thiazolyl-C_4_-H), 7.44 (d, *J* = 7.5 Hz, 2H, dichloroaniline-C_3,5_-H), 7.54 (d, *J* = 8.0 Hz, 2H, sulfamoylphenyl-C_3,5_-H), 7.87 (d, *J* = 8.0 Hz, 2H, sulfamoylphenyl-C_2,6_-H), 9.40 (s, 1H, ph-NH, D_2_O exchangeable), 10.82 (s, 1H, CONH, D_2_O exchangeable), 12.04 (s, 1H, sulfamoyl NH, D_2_O exchangeable). ^13^C-NMR (125 MHz, DMSO-d_6_) δ 10.44 (triazolyl-5-CH_3_), 14.22 (N=C-CH_3_), 25.72 (O=C-CH_2_), 108.62 (thiazolyl-C_5_), 116.91 (substituted aniline-C_6_), 119.76 (sulfamoylphenyl-C_3, 5_), 122.27 (substituted aniline-C_4_), 123.45 (dichloroaniline-C_4_), 125.15 (substituted aniline-C_5_), 126.05 (thiazolyl-C_4_), 127.15 (substituted aniline-C_2_), 128.86 (substituted aniline-C_3_), 129.46 (sulfamoylphenyl-C_2,6_), 129.82 (dichloroaniline-C_2,6_), 130.25 (dichloroaniline-C_3,5_), 133.51 (triazolyl-C_5_), 136.11 (sulfamoylphenyl-C_4_), 136.64 (sulfamoylphenyl-C_1_), 139.28 (N=C), 142.19 (triazolyl-C_4_), 143.49 (dichloroaniline-C_1_), 144.12 (substituted aniline-C_1_), 168.62 (thiazolyl-C_2_), 170.43 (C=O) Anal. Calcd for C_28_H_24_Cl_2_N_8_O_3_S_2_ (655.57): C, 51.30; H, 3.69; N, 17.09; S, 9.78. Found: C, 51.42; H, 3.73; N, 17.01; S, 9.71.

**4-(4-(1-(2-(2-(2-((2,6-Dichlorophenyl)amino)phenyl)acetyl)hydrazineylidene)ethyl)-5-methyl-1H-1,2,3-triazol-1-yl)-N-(pyrimidin-2-yl)benzenesulfonamide (6f)**

Off-white crystals; yield: 49%, m.p.: 276–278 °C. IR (KBr, cm^−1^): 3278, 3263 and 3224 (NH), 1620 (C=O), 1508 (C=N), 1297, 1164 (SO_2_). ^1^H-NMR (400 MHz, DMSO-d_6_) δ 2.42 (s, 3H, N=C-CH_3_), 2.68 (s, 3H, triazolyl-5-CH_3_), 3.52 (s, 2H, O=C-CH_2_), 6.39 (dd, *J* = 7.5, 1.9 Hz, 1H, substituted aniline-C_6_-H), 6.74 (ddd, *J* = 7.5, 1.9, 1.4 Hz 1H, substituted aniline-C_4_-H), 6.91 (ddd, *J* = 7.5, 1.9, 1.4 Hz, 1H, substituted aniline-C_5_-H), 7.03–7.07 (m, 2H, pyrimidinyl-C_5_-H and dichloroaniline-C_4_-H), 7.16 (d, *J* = 7.5 Hz, 1H, substituted aniline-C_3_-H), 7.44 (d, *J* = 7.5 Hz, 2H, dichloroaniline-C_3,5_-H), 7.63 (d, *J* = 8.0 Hz, 2H, sulfamoylphenyl-C_3,5_-H), 7.80 (d, *J* = 8.0 Hz, 2H, sulfamoylphenyl-C_2,6_-H), 8.58 (d, *J* = 4.9 Hz, 2H, pyrimidinyl-C_4,6_-H), 9.42 (s, 1H, ph-NH, D_2_O exchangeable), 10.64 (s, 1H, CONH, D_2_O exchangeable), 12.10 (s, 1H, sulfamoyl NH, D_2_O exchangeable). ^13^C-NMR (125 MHz, DMSO-d_6_) δ 10.43(triazolyl-5-CH_3_), 14.22(N=C-CH_3_), 25.53(O=C-CH_2_), 114.74(pyrimidinyl-C_5_), 116.91(substituted aniline-C_6_), 120.83(sulfamoylphenyl-C_3,5_), 123.25(substituted aniline-C_4_), 123.92(dichloroaniline-C_4_), 125.45(substituted aniline-C_5_), 127.82(substituted aniline-C_2_), 129.09(substituted aniline-C_3_), 129.23(sulfamoylphenyl-C_2,6_), 129.42(dichloroaniline-C_2,6_), 130.60(dichloroaniline-C_3,5_), 134.69(triazolyl-C_5_), 136.88(sulfamoylphenyl-C_4_), 136.93(sulfamoylphenyl-C_1_), 139.37(N=C), 142.09(triazolyl-C_4_), 143.35(dichloroaniline-C_1_), 143.60(substituted aniline-C_1_), 156.41(pyrimidinyl-C_4,6_), 157.23(pyrimidinyl-C_2_), 170.38(C=O). Anal.Calcd for C_29_H_25_Cl_2_N_9_O_3_S (650.54): C, 53.54; H, 3.87; N, 19.38; S, 4.93. Found: C, 53.62; H, 3.98; N, 19.42; S, 4.85. EIMS *m*/*z* (% relative abundance): 652.91 (19.86) (M^+•^ + 2), 649.98 (59.58) (M^+•^), 540.56 (46.91), 510.93 (67.01), 347.20 (47.21), 261.34 (52.49), 77.32 (100) (base peak), 49.51 (64.50).

**4-(4-(1-(2-(2-Hydroxybenzoyl)hydrazineylidene)ethyl)-5-methyl-1H-1,2,3-triazol-1-yl)benzenesulfonamide (6g)**

White crystals; yield: 56%, m.p.: 220–222 °C. IR (KBr, cm^−1^): 3447 (OH), 3348, 3309 (NH_2_), 3211 (NH), 1618 (C=O), 1593 (C=N), 1359, 1122 (SO_2_). ^1^H-NMR (500 MHz, DMSO-d6) δ 2.56 (s, 3H, N=C-CH_3_), 2.68 (s, 3H, triazolyl-5-CH_3_), 7.00 (t, 1H, *J* = 7.8Hz, salicyl-C_5_-H), 7.04 (d, 1H, *J* = 7.8Hz, salicyl-C_3_), 7.44 (td, *J* = 7.8, 1.5 Hz, 1H, salicyl-C_4_-H), 7.61 (s, 1H, NH_2_, D_2_O exchangeable), 7.91 (d, *J* = 8.0 Hz, 2H, sulfamoylphenyl-C_3,5_-H), 7.99 (dd, *J* = 7.8, 1.5 Hz, 1H, salicyl-C_6_-H), 8.07 (d, *J* = 8.0 Hz, 2H, sulfamoylphenyl-C_2,6_-H), 11.38 (s, 1H, CONH, D_2_O exchangeable), 11.81 (s, 1H, OH, D_2_O exchangeable). Anal.Calcd for C_18_H_18_N_6_O_4_S (414.44): C, 52.17; H, 4.38; N, 20.28; S, 7.74. Found: C, 52.28; H, 4.46; N, 20.17; S, 7.84.

**4-(4-(1-(2-(2-Hydroxybenzoyl)hydrazineylidene)ethyl)-5-methyl-1H-1,2,3-triazol-1-yl)-N-(thiazol-2-yl)benzenesulfonamide (6h)**

White crystals; yield: 62%, m.p.: 227–229 °C. IR (KBr, cm^−1^): 3425 (OH), 3264 (NH), 1650 (C=O), 1597 (C=N), 1282, 1149 (SO_2_). ^1^H-NMR (500 MHz, DMSO-d6) δ 2.34 (s, 3H, N=C-CH_3_), 2.67 (s, 3H, triazolyl-5-CH_3_), 6.93 (br.s, 1H, thiazolyl-C_5_-H), 7.00 (t, 1H, *J* = 7.8 Hz, salicyl-C_5_-H),7.04 (d, 1H, *J* = 7.8 Hz, salicyl-C_3_), 7.23 (s, 1H, thiazolyl-C_4_-H), 7.43 (td, *J* = 7.8, 1.5 Hz, 1H, salicyl-C_4_-H), 7.89 (d, *J* = 8.0 Hz, 2H, sulfamoylphenyl-C_3,5_-H), 7.99 (d, *J* = 7.8 Hz, 1H, salicyl-C_6_-H), 8.21 (d, *J* = 8.0 Hz, 2H, sulfamoylphenyl-C_2,6_-H), 11.37 (s, 1H, CONH, D_2_O exchangeable), 11.80 (s, 1H, OH, D_2_O exchangeable), 12.41 (s, 1H, sulfamoyl NH, D_2_O exchangeable). ^13^C-NMR (125 MHz, DMSO-d_6_) δ 9.25(triazolyl-5-CH_3_), 17.29 (N=C-CH_3_), 111.64 (thiazolyl-C_5_), 117.67 (salicyl-C_1_), 119.62 (salicyl-C_3_), 122.53 (sulfamoylphenyl-C_3,5_), 126.34 (salicyl-C_5_), 128.28 (thiazolyl-C_4_), 133.07 (sulfamoylphenyl-C_2,6_), 136.60 (salicyl-C_2_), 139.16 (salicyl-C_4_), 143.95 (triazolyl-C_5_), 146.52 (sulfamoylphenyl-C_4_), 152.90 (sulfamoylphenyl-C_1_), 156.08 (triazolyl-C_4_), 159.96 (N=C), 164.74 (salicyl-C_6_), 167.31 (C=O), 171.75 (thiazolyl-C_2_). Anal.Calcd for C_21_H_19_N_7_O_4_S_2_ (497.55): C, 50.69; H, 3.85; N, 19.71; S, 12.89. Found: C, 50.75; H, 3.93; N, 19.60; S, 12.93.

**4-(4-(1-(2-(2-Hydroxybenzoyl)hydrazineylidene)ethyl)-5-methyl-1H-1,2,3-triazol-1-yl)-N-(pyrimidin-2-yl)benzenesulfonamide (6i)**

Off-white crystals; yield: 69%, m.p.: 248–250 °C. IR (KBr, cm^−1^): 3438 (OH), 3306 (NH), 1610 (C=O), 1570 (C=N), 1322, 1165 (SO_2_). ^1^H-NMR (500 MHz, DMSO-d_6_) δ 2.41 (s, 3H, N=C-CH_3_), 2.58 (s, 3H, triazolyl-5-CH_3_), 6.92–6.94 (m, 2H, salicyl-C_3,5_-H), 7.03 (t, *J* = 5.0 Hz, 1H, pyrimidinyl-C_5_-H),7.39–7.42 (m, salicyl-C_4_-H), 7.83 (d, *J* = 8.0 Hz, 2H, sulfamoylphenyl-C_3,5_-H), 8.15–8.17 (m, 3H, salicyl-C_6_-H and sulfamoylphenyl-C_2,6_-H), 8.49 (d, *J* = 5.0 Hz, 2H, pyrimidinyl-C_4,6_-H), 11.10 (s, 1H, CONH, D_2_O exchangeable), 11.60 (s, 1H, OH, D_2_O exchangeable), 12.04 (s, 1H, sulfamoyl NH, D_2_O exchangeable). ^13^C-NMR (125 MHz, DMSO-D_6_) δ 9.86(triazolyl-5-CH_3_), 21.11(N=C-CH_3_), 115.67 (pyrimidinyl-C_5_), 118.22(salicyl-C_1_), 119.46(salicyl-C_3_), 125.76(sulfamoylphenyl-C_3,5_), 129.09(salicyl-C_5_), 129.67(sulfamoylphenyl-C_2,6_),134.85(salicyl-C_2_), 138.08(salicyl-C_4_), 141.93(triazolyl-C_5_), 143.06(sulfamoylphenyl-C_4_), 147.29(sulfamoylphenyl-C_1_), 156.66(triazolyl-C_4_), 158.45(N=C), 164.57 (pyrimidinyl-C_4,6_), 167.38(salicyl-C_6_), 172.10 pyrimidinyl-C_2_), 193.39 (C=O). Anal.Calcd for C_22_H_20_N_8_O_4_S (492.51): C, 53.65; H, 4.09; N, 22.75; S, 6.51. Found: C, 53.73; H, 4.20; N, 22.67; S, 6.55.

**4-(4-(1-(2-(2-Hydroxybenzoyl)hydrazineylidene)ethyl)-5-methyl-1H-1,2,3-triazol-1-yl)-N-(4-methylpyrimidin-2-yl)benzenesulfonamide (6j)**

Buff crystals; yield: 60%, m.p.: 261–263 °C. IR (KBr, cm^−1^): 3511 (OH), 3280 (NH), 1635 (C=O), 1562 (C=N), 1298, 1148 (SO_2_). ^1^H-NMR (500 MHz, DMSO-d6) δ 1.91 (s, 3H, N=C-CH_3_), 2.34 (s, 3H, pyrimidinyl-4-CH_3_), 2.67 (s, 3H, triazolyl-5-CH_3_), 6.93 (s, 1H, pyrimidinyl-C_5_-H), 7.00 (t, 1H, *J* = 7.8 Hz, salicyl-C_5_-H),7.04 (br.s, 1H, *J* = 7.8 Hz, salicyl-C_3_), 7.43 (t, *J*= 7.8Hz, 1H, salicyl-C_4_-H), 7.89 (d, *J* = 8.0 Hz, 2H, sulfamoylphenyl-C_3,5_-H), 7.99 (d, *J* = 7.8 Hz, 1H, salicyl-C_6_-H), 8.21 (d, *J* = 8.0 Hz, 2H, sulfamoylphenyl-C_2,6_-H), 8.36 (s, 1H, pyrimidinyl-C_6_-H), 11.37 (s, 1H, CONH, D_2_O exchangeable), 11.80 (s, 1H, OH, D_2_O exchangeable), 12.48 (s, 1H, sulfamoyl NH,D_2_O exchangeable). Anal.Calcd for C_23_H_22_N_8_O_4_S (506.54): C, 54.54; H, 4.38; N, 22.12; S, 6.33. Found: C, 54.66; H, 4.49; N, 22.01; S, 6.43. EIMS *m*/*z* (% relative abundance): 506.32 (26.80) (M^+•^), 496.14 (44.14), 413.41 (43.83), 185.26 (80.30), 151.34 (66.81), 107.37 (67.56), 69.03 (100) (base peak).

### 3.2. Biological Screening

#### 3.2.1. In Vitro Human COX-1 and COX-2 Enzymatic Inhibitory Activities 

In vitro human COX-1 and human COX-2 enzymatic inhibitory activity of the tested compounds as well as the reference drugs was carried out according to the previously mentioned procedures [22,23]; for details, see Page S15, Supplementary File.

#### 3.2.2. Carrageenan-Induced Paw Edema in Mice

In vivo carrageenan-induced paw edema bioassay in mice was implemented on the three analogue **6b**, **6j** and **6e** as well as the reference drugs celecoxib and diclofenac, as previously reported [24,25] (approved by HU-IACUC) [41]. For more details, see Pages S15 and S16, Supplementary File. 

#### 3.2.3. Determination of ED_50_

Dose–response curves of the compounds **6b, 6e** and **6j** as well as the reference drugs celecoxib and diclofenac were plotted and their ED_50_ values were calculated, as previously reported [9] (approved by HU-IACUC) [41]. For more details, see Page S16, Supplementary File.

#### 3.2.4. Estimation of Rat Serum Prostaglandin E2 (PGE2)

The quantitative determination of PGE2 in biological fluids was carried out for compounds **6b, 6j** and **6e** as well as the reference drugs celecoxib and diclofenac according to the competitive immunoassay procedure, as previously reported [42] (approved by HU-IACUC) [41]. For more details, see Page S16, Supplementary File.

#### 3.2.5. Ulcerogenic Effects

The ulcerogenic effect of the tested compounds was evaluated taking celecoxib and diclofenac as reference standards and was carried out according to the reported procedures [43] (approved by HU-IACUC) [41]. For more details, see Page S16, Supplementary File.

### 3.3. Molecular Docking Studies

Molecular docking studies were performed as previously reported [44]. For more details see Pages S16 and S17, Supplementary File.

### 3.4. In Silico Prediction of the Physicochemical Properties, Drug-Likeness Score, Pharmacokinetics, Toxicity Profile and Ligand Efficiency Metrics

In the present study, the Molinspiration chemoinformatic server was utilized for the prediction of the physicochemical properties, the Osiris property explorer for calculating drug-likeness scores and the Pre-ADMET calculator for the toxicological effects and pharmacokinetics.

## 4. Conclusions

The fundamental target of the present study was to design selective COX-2 inhibitors with high safety profiles. The target compounds were designed based on a pharmacophoric hybridization strategy through combining the COX-2 inhibitor pharmacophores, benzenesulfonamide derivatives, the 1,2,3-triazole moiety and traditional NSAIDs into one molecule with more potentiated and selective COX-2 inhibitory properties. 

The newly synthesized compounds were evaluated for their in vitro COX-1/2 inhibitory activity. Compounds **6b**, **6e** and **6j** revealed the highest COX-2 inhibitory activity among the series, with IC_50_ values of 0.04 µm, 0.05 µm and 0.04 µm, respectively, and SI values of 329, 204 and 312, respectively. 

Moreover, the highly active COX-2 inhibitors **6b**, **6e** and **6j** were screened in vivo for their expected anti-inflammatory properties. Interestingly, compounds **6b** and **6j** exhibited significant inhibition of carrageenan-induced paw edema in mice, showing a higher % of protection from edema (99.45% and 91.21%, respectively) than that of celecoxib (89.01%). Additionally, their ED_50_ values (11.74 and 13.38 µmol/kg, respectively) were lower than that of celecoxib (16.24 µmol/kg). 

Measuring the plasma levels of PGE2 in rats demonstrated the potential of the three compounds to inhibit PGE2 production, showing a % inhibition of 90.70% and 86.34%, respectively, which emphasized their COX-2 inhibitory potential. Moreover, compounds **6b** and **6j** exhibited a gastric safety profile comparable to celecoxib. 

Furthermore, molecular docking studies of both compounds into the active site of the COX-2 isozyme proved their potential COX-2 inhibitory activity. Interestingly, docking results were consistent with their IC_50_ values and selectivity indices according to the in vitro testing data results (Table 1). 

In addition, in silico calculation of the pharmacokinetic and toxicity properties (ADMET) of compounds **6b** and **6j** suggested their capacity to act orally with an expected high safety profile. Finally, LE values of both compounds regarding their COX-2 inhibitory activities were consistent with the acceptable LE value for lead compounds (around 0.3). 

To conclude, the overall PGE2 and COX-2 inhibitory profiles revealed by the newly synthesized compounds **6b** and **6j** confirmed their in vitro and in vivo potent and selective COX-2 inhibition. These lead compounds deserve further derivatization to optimize their pharmacokinetic and pharmacodynamic profiles. 

## Figures and Tables

**Table 1:** In vitro human COX-1/COX-2 enzymatic inhibitory activities and COX selectivity indices of the newly synthesized compounds.

Compound NO.	COX-1	COX-2	SI ^c^
	IC_50_ (µM) ^a^	IC_50_ (µM) ^b^	COX-1/COX-2
**Celecoxib**	14.7 ± 0.0577	0.05 ± 0.0003	294
**Indomethacin**	0.1 ± 0.0033	0.48 ± 0.0058	0.21
**Diclofenac sodium**	3.8 ± 0.0333	0.84 ± 0.0033	4.52
**1c**	5.93 ± 0.0882	0.09 ± 0.0006	65.89
**1d**	4.6 ± 0.1155	0.1 ± 0.0033	46
**4c**	8.23 ± 0.0667	0.09 ± 0.0003	91.44
**4d**	9.27 ± 0.0667	0.11 ± 0.0057	84.27
**6a**	9.13 ± 0.0882	0.08 ± 0.0006	114.13
**6b**	13.17 ± 0.1202	0.04 ± 0.0007	329.25
**6c**	11.17 ± 0.0333	0.07 ± 0.0006	159.57
**6d**	8.4 ± 0.0577	0.08 ± 0.0009	105
**6e**	10.23 ± 0.0333	0.05 ± 0.0006	204.6
**6f**	12.67 ± 0.0333	0.08 ± 0.0007	158.38
**6g**	6.67 ± 0.0667	0.08 ± 0.0006	83.38
**6h**	8.27 ± 0.0667	0.09 ± 0.0006	91.89
**6i**	10.23 ± 0.0882	0.07 ± 0.0006	146.14
**6j**	12.5 ± 0.0577	0.04 ± 0.0006	312.5

**^a,b^** Concentration of the compound that causes 50% inhibition of enzymatic activity of COX-1 or COX-2. **^a,b^** All values are expressed as mean ± SEM of triplicate determinations. **^c^** COX-2 selectivity index: (COX-1 _IC50_/COX-2 _IC50_).

**Table 2:** Effects of the tested compounds **6b**, **6e** and **6j** on carrageenan-induced paw edema in mice (mm) *; percentage inhibition of paw edema and ED_50_ values (µmol/kg).

Compound No. ^a^	Thickness of Edema (mm) ^b^					ED_50_^f^ (µmol/kg) (95% Confidence Level)
	0 h	2 h	4 h	6 h	8 h	
**Control** **(carrageenan)**	2.13 ± 0.006	3.64 ± 0.007 ^d^	3.77 ± 0.006 ^d^	3.86 ± 0.007 ^d^	3.85 ± 0.009 ^d^	
**Celecoxib**	2.10 ± 0.005	2.54 ± 0.007 ^c^ (70.86%) ^e^	2.44 ± 0.011 ^c^ (79.27%)	2.32 ± 0.010 ^c^ (87.28%)	2.30 ± 0.009 ^c^ (89.01%)	16.24 (14.09–18.60)
**Diclofenac**	2.08 ± 0.006	2.96 ± 0.009 ^c,d^ (41.72%)	2.81 ± 0.009 ^c,d^ (55.49%)	2.64 ± 0.01 ^c,d^ (67.63%)	2.58 ± 0.01 ^c,d^ (72.53%)	18.35 (16.22–20.63)
**6b**	2.14 ± 0.006	2.34 ± 0.009 ^c,d^ (86.75%)	2.23 ± 0.009 ^c,d^ (94.51%)	2.18 ± 0.009 ^c,d^ (97.69%)	2.15 ± 0.007 ^c,d^ (99.45%)	11.74 (10.46–13.16)
**6e**	2.12 ± 0.007	2.75 ± 0.008 ^c,d^ (58.28%)	2.63 ± 0.010 ^c,d^ (68.90%)	2.54 ± 0.009 ^c,d^ (75.72%)	2.52 ± 0.006 ^c,d^ (78.02%)	18.23 (16.61–19.92)
**6j**	2.08 ± 0.007	2.48 ± 0.005 ^c,d^ (73.51%)	2.37 ± 0.009 ^c,d^ (82.32%)	2.28 ± 0.011 ^c,d^ (88.44%)	2.24 ± 0.009 ^c,d^ (91.21%)	13.38 (12.04–14.84)

* Data were analyzed by one-way ANOVA followed by Tukey–Kramer post hoc test for multiple comparisons. **^a^** Dose level for all compounds, po: 10 µmol/kg b.wt. **^b^** Values are expressed as mean ± SEM (number of animals n = 5 mice). **^c^** Means are significantly different from the control group at *p* < 0.0001. **^d^** Means are significantly different from the celecoxib group at *p* < 0.0001. **^e^** Values between parentheses: percentage anti-inflammatory activity (AI%). **^f^** ED_50_, median effective dose in µmol/kg; the dose that exerts an effect in 50% of the population (95% confidence level).

**Table 3:** Rat serum prostaglandin E2 of the targeted compounds (pg/mL) * and % inhibition of PGE2.

Compound NO. ^a^	PGE2 Serum Conc. (pg/mL) ^b^	% Inhibition
**Control (pre-carrageenan)**	116.450 ± 5.47 ^c,d^	-
**Control 1 (post-carrageenan)**	735.470 ± 8.03 ^d^	-
**Celecoxib**	157.245 ± 7.33 ^c,d^	78.62%
**Diclofenac**	203.973 ± 6.35 ^c,d^	72.27%
**6b**	68.388 ± 7.13 ^c,d^	90.70%
**6e**	185.329 ± 6.79 ^c,d^	74.80%
**6j**	100.435 ± 5.66 ^c,d^	86.34%

* Data were analyzed by one-way ANOVA followed by the Tukey–Kramer post hoc test for multiple comparisons. **^a^** Dose level for all compounds, po: 10 µmol/kg b.wt. **^b^** Values are expressed as mean ± SEM (number of animals n = 5 mice). ^c^ Means are significantly different from the control group 1 (*p* < 0.0001). ^d^ Means are significantly different from each other (*p* < 0.0001).

**Table 4:** In silico prediction of the physicochemical properties, drug-likeness, pharmacokinetics and ligand efficiency metrics of compounds **6b** and **6j**.

	6b	6j	Celecoxib	Diclofenac
**Physicochemical Parameters and Drug-Likeness**				
**Log P** ^**a**^	4.67	2.53	3.61	4.57
**M.Wt** ^**b**^	565.73	506.55	381.38	296.15
**HBA** ^**c**^	10	12	5	3
**HBD** ^**d**^	2	3	2	2
**Lipinski’s violation ** ^**e**^	1	2	0	0
**NROTB** ^**f**^	10	7	4	4
**TPSA** ^**g**^	131.24	164.36	77.99	49.33
**%ABS** ^**h**^	63.72	52.30	82.09	91.98
**Volume** ^**i**^	493.62	423.19	298.65	238.73
**S** ^**j**^	0.011	4.81	2.08	6.17
**Drug-likeness score ** ^**k**^	0.16	0.25	0.37	0.38
**Pharmacokinetics (ADME)**				
**Caco-2** ^**l**^	11.54	0.56	0.49	24.53
**MDCK** ^**m**^	0.052	2.704	45.05	51.46
**HIA** ^**n**^	96.59	91.53	96.69	95.96
**BBB** ^**o**^	0.125	0.049	0.027	1.39
**PPB** ^**p**^	87.67	97.87	91.08	91.96
**Ligand efficiency metrics**				
**LE (COX-2)** ^**q**^	0.26	0.28	0.38	0.44
**LLE** ^**r**^	2.73	4.87	2.87	1.51

**^a^** n-Octanol and water partition coefficient. **^b^** Molecular weight. **^c^** Number of H-bond acceptors. **^d^** Number of H-bond donors. **^e^** Lipinski’s rule of 5 violations (log *p* ≤ 5, M.Wt. ≤ 500, HBA ≤ 10 and HBD ≤ 5). **^f^** Number of rotatable bonds (Veber’s criteria ≤ 10). **^g^** Topological polar surface area (Veber’s criteria ≤ 140 Å^2^). **^h^** Percentage of absorption = 109 − (0.345 × TPSA). **^i^** Molecular volume. **^j^** Solubility in mg/L. **^k^** Drug-likeness score. **^l^** Caco-2 permeability (nm/s): permeability through cells derived from human colon adenocarcinoma; <4 nm/s (low permeability), values from 4 to 70 nm/s (medium permeability) and > 70 nm/sec (high permeability). Permeability through human colon adenocarcinoma cells. **^m^** MDCK permeability (nm/sec): permeability through Madin–Darby canine kidney cells; <25 nm/s (low permeability), values from 25 to 500 nm/s (medium permeability) and >500 nm/s (high permeability). **^n^** Human intestinal absorption percentage: values from 0 to 20% (poorly absorbed), values from 20 to 70% (moderately absorbed) and values from 70 to 100% (well-absorbed). **^o^** Blood–brain barrier penetration coefficient: values < 0.1 (low CNS penetration), values from 0.1 to 2 (medium CNS absorption) and values > 2 (high CNS absorption). **^p^** Plasma protein binding: values < 90% (poorly bound) and > 90% (strongly bound). **^q^** Ligand efficiency = (pIC_50_x1.37)/non-hydrogen atoms. **^r^** Lipophilic ligand efficiency = pIC_50_ − logP.

## Data Availability

Data are contained within the article and supplementary material.

## Supplementary Materials

The following supporting information can be downloaded at: https://www.mdpi.com/article/10.3390/ph15101165/s1. Figure S1: ^1^H-NMR spectrum of (**1c**); Figure S2: ^1^H-NMR spectrum of (**1d**); Figure S3: ^1^H-NMR spectrum of (**4c**); Figure S4: ^1^H-NMR spectrum of (**4d**); Figure S5: ^1^H-NMR spectrum of (**6a**); Figure S6: ^1^H-NMR spectrum of (**6b**); Figure S7: ^1^H-NMR spectrum of (**6c**); Figure S8: ^1^H-NMR spectrum of (**6d**); Figure S9: ^1^H-NMR spectrum of (**6e**); Figure S10: ^1^H-NMR spectrum of (**6f**); Figure S11: ^1^H-NMR spectrum of (**6g**); Figure S12: ^1^H-NMR spectrum of (**6h**); Figure S13: ^1^H-NMR spectrum of (**6i**); Figure S14: ^1^H-NMR spectrum of (**6j**); Figure S15: ^13^C-NMR spectrum of (**1c**); Figure S16: ^13^C-NMR spectrum of (**4d**); Figure S17: ^13^C-NMR spectrum of (**6a**); Figure S18: ^13^C-NMR spectrum of (**6b**); Figure S19: ^13^C-NMR spectrum of (**6e**); Figure S20: ^13^C-NMR spectrum of (**6f**); Figure S21: ^13^C-NMR spectrum of (**6h**); Figure S22: ^13^C-NMR spectrum of (**6i**); Figure S23: EI-MS spectrum of (**6b**); Figure S24: EI-MS spectrum of (**6f**); Figure S25: EI-MS spectrum of (**6j**).
