# Supplementary material for: Rational Design and Synthesis of New Selective COX-2 Inhibitors with In Vivo PGE2-Lowering Activity by Tethering Benzenesulfonamide and 1,2,3-Triazole Pharmacophores to Some NSAIDs
Source: Pharmaceuticals (Basel). 2022 Sep 20;15(10):1165. doi: 10.3390/ph15101165 (PMC9609428; doi:10.3390/ph15101165)
Supplement: Supplementary file 1 [file pharmaceuticals-15-01165-s001.zip › pharmaceuticals-1900933-supplementary.pdf]

Rational design and synthesis of new selective COX-2 inhibitors with *in vivo* PGE2-lowering activity by tethering benzenesulfonamide and 1,2,3-triazole pharmacophores to some NSAIDs

Experimental

Spectral data

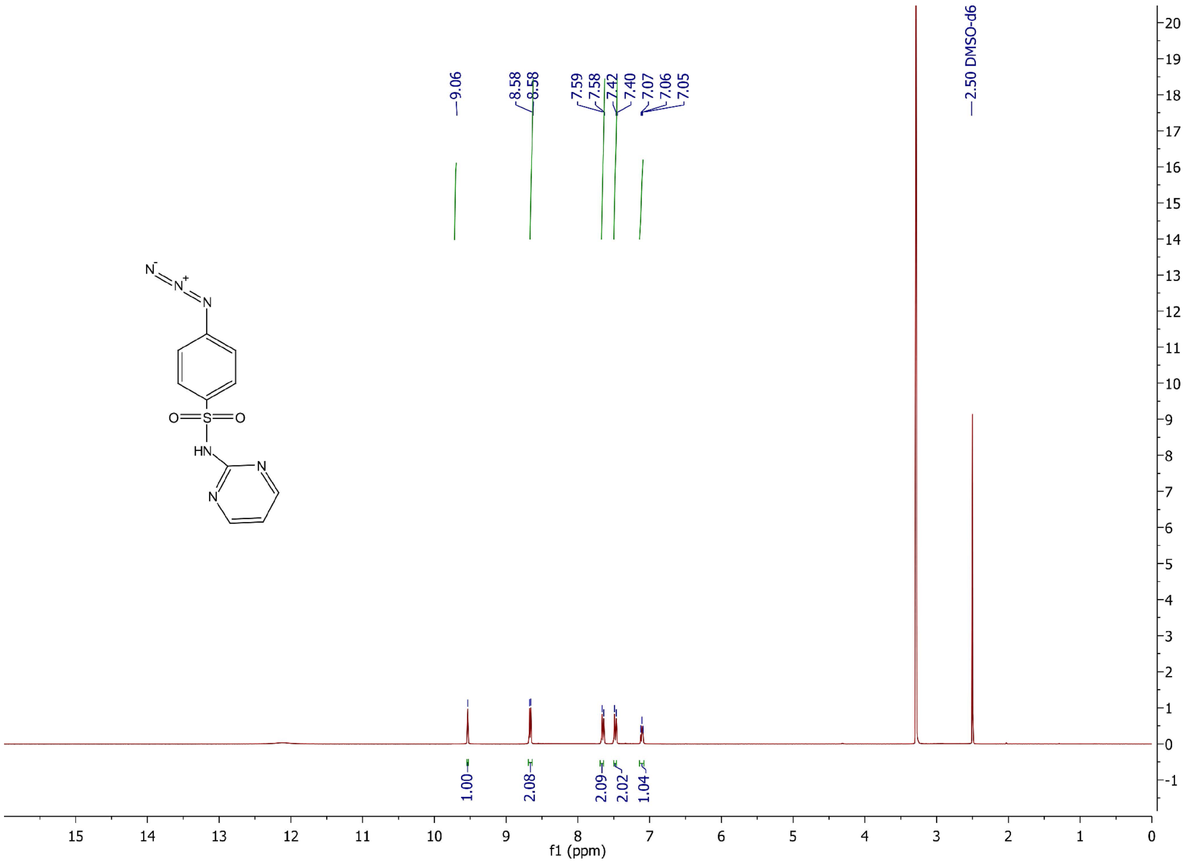

Figure S1. ^{1}**H-NMR spectrum of compound 1c (DMSO-*d*$_6$)**

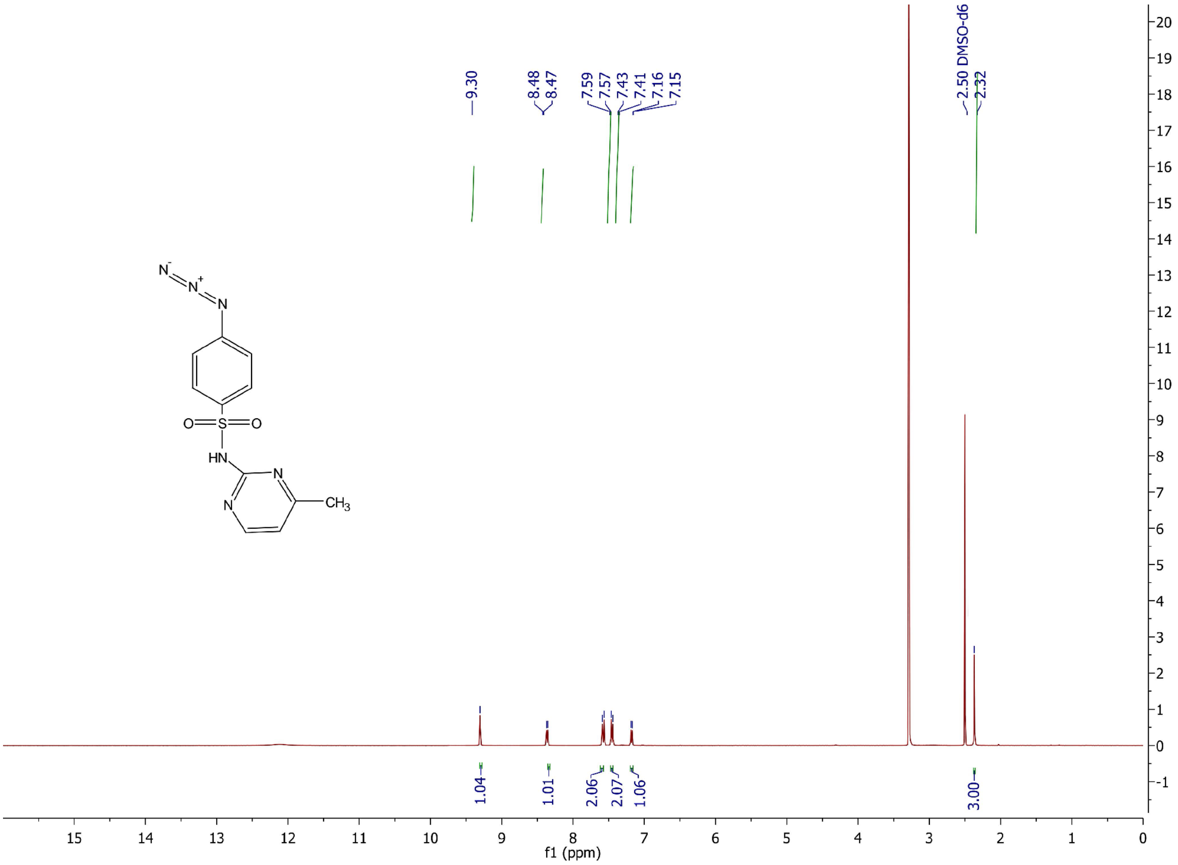

Figure S2. ^{1}H-NMR spectrum of compound 1d (DMSO-*d$_6$*)

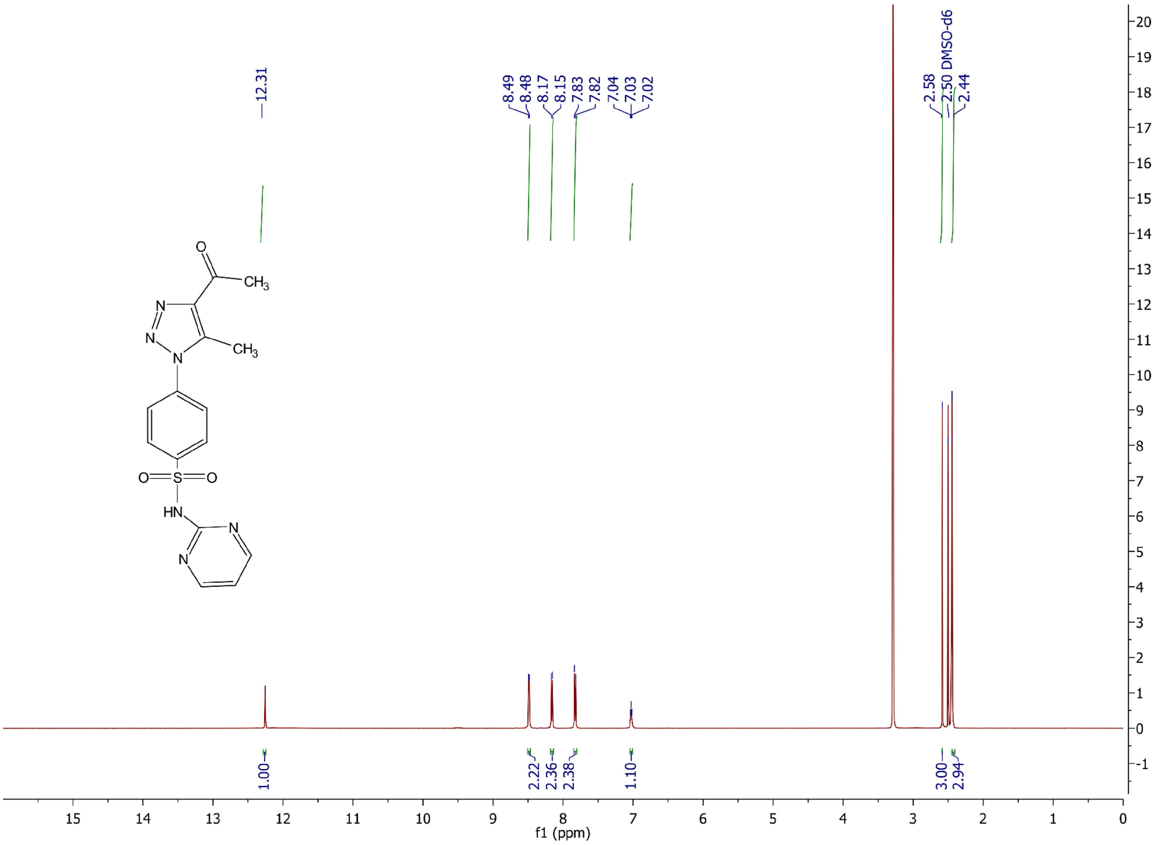

Figure S3. ^{1}H-NMR spectrum of compound 4c (DMSO-*d$_6$*)

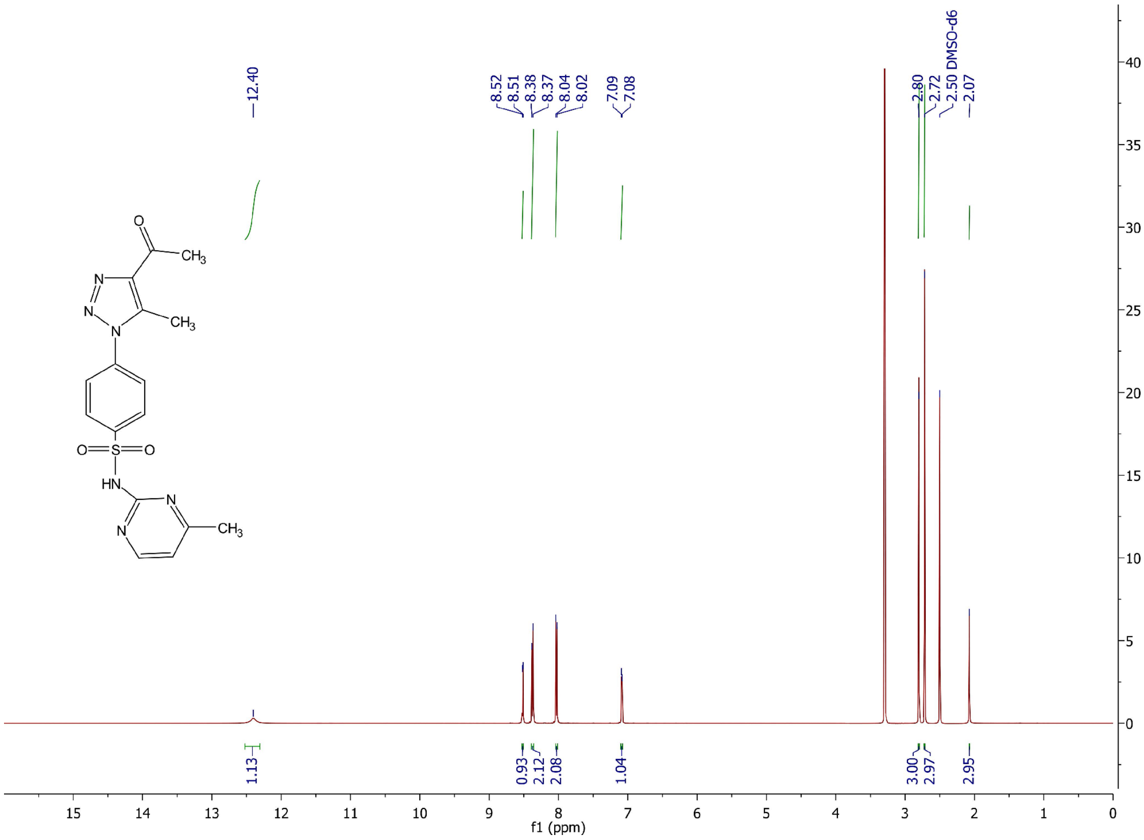

Figure S4. ^{1}H-NMR spectrum of compound 4d (DMSO-d_6)

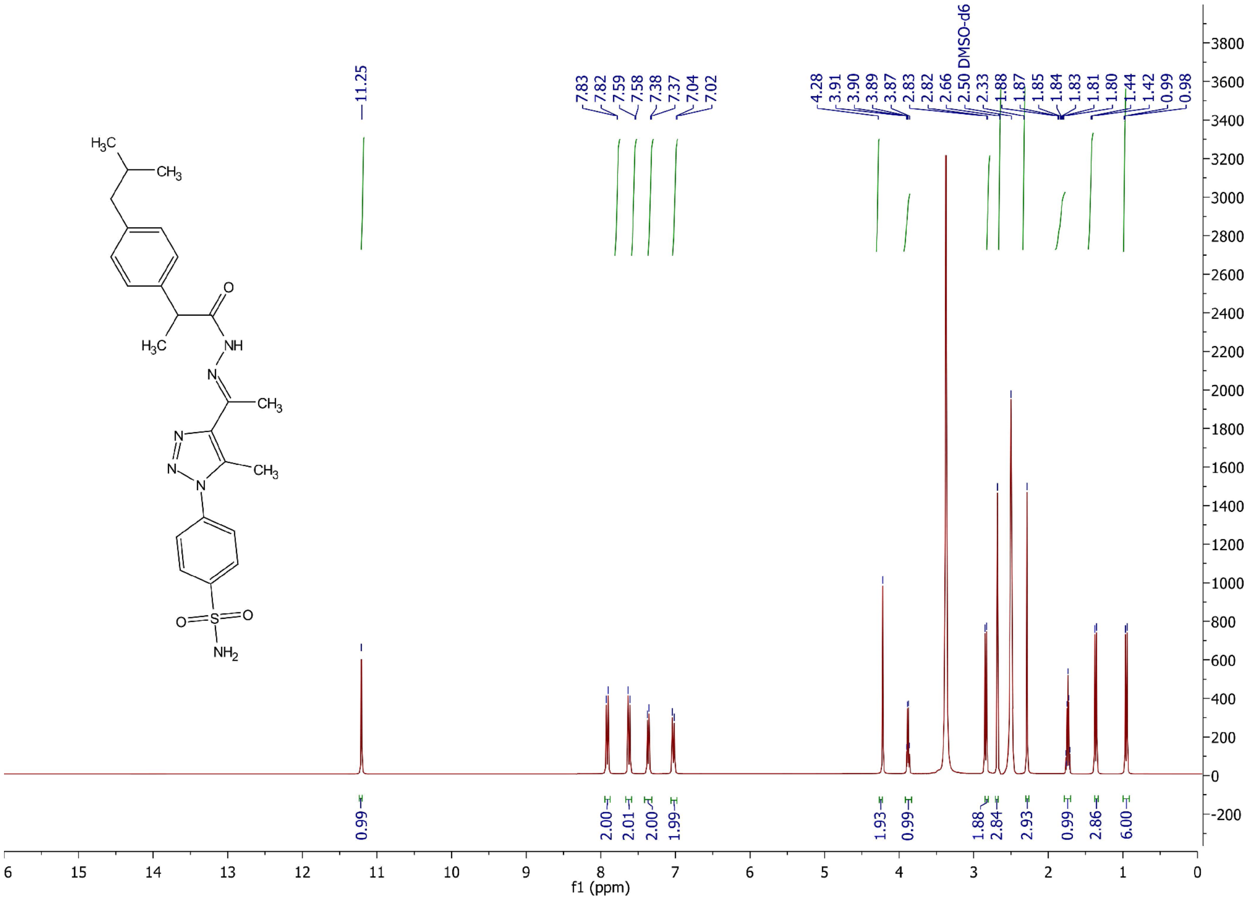

Figure S5. ¹H-NMR spectrum of compound 6a (DMSO-d_6)

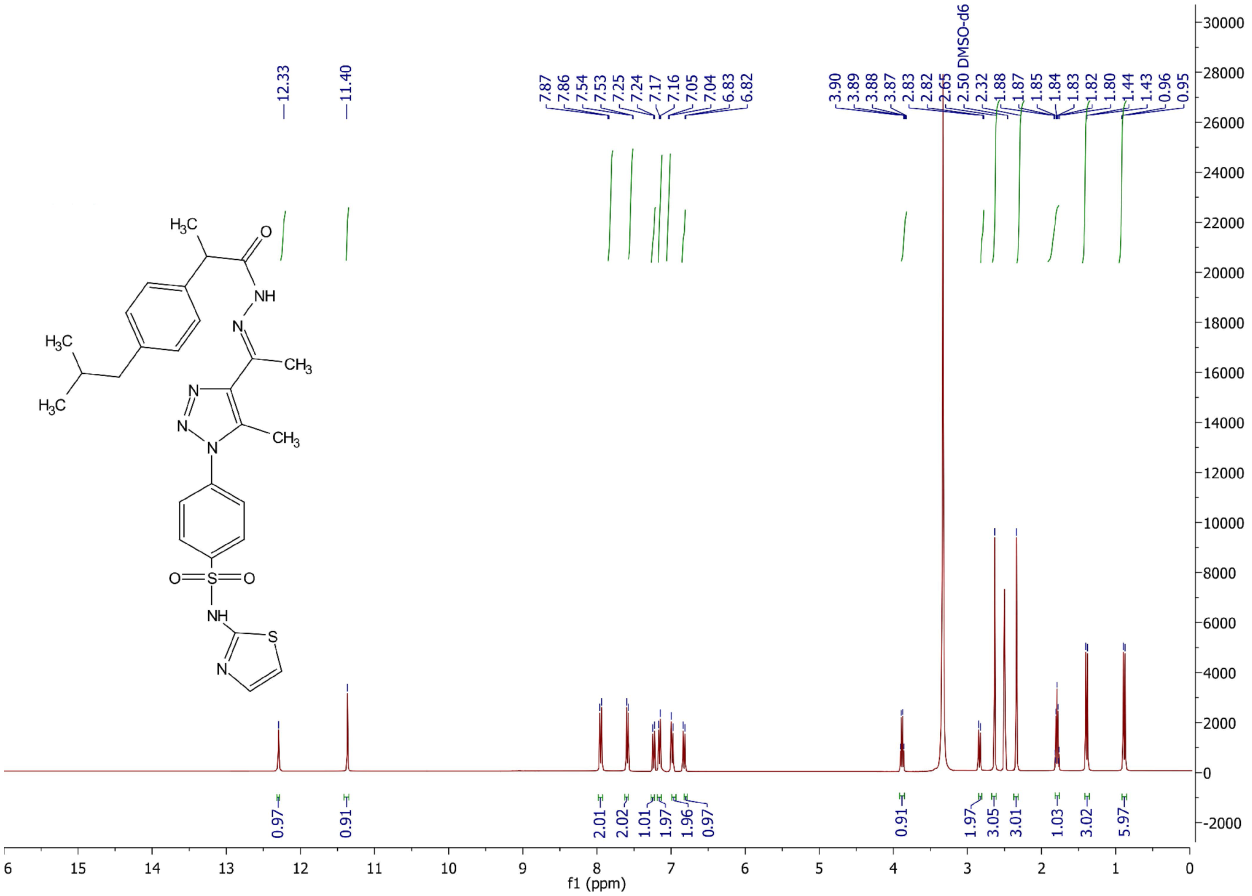

Figure S6. ¹H-NMR spectrum of compound 6b (DMSO-d_6)

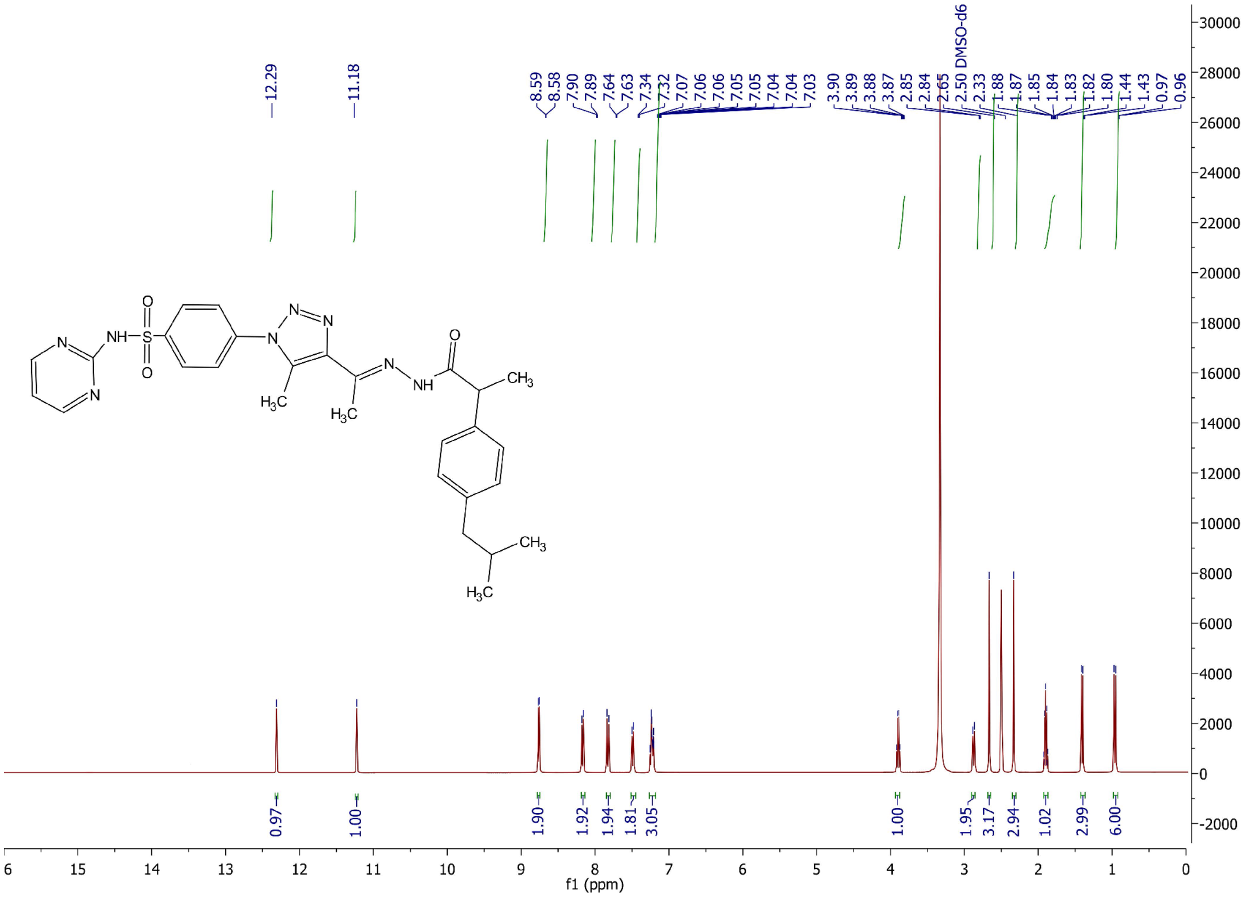

Figure S7. ^{1}H-NMR spectrum of compound 6c (DMSO-*d₆*)

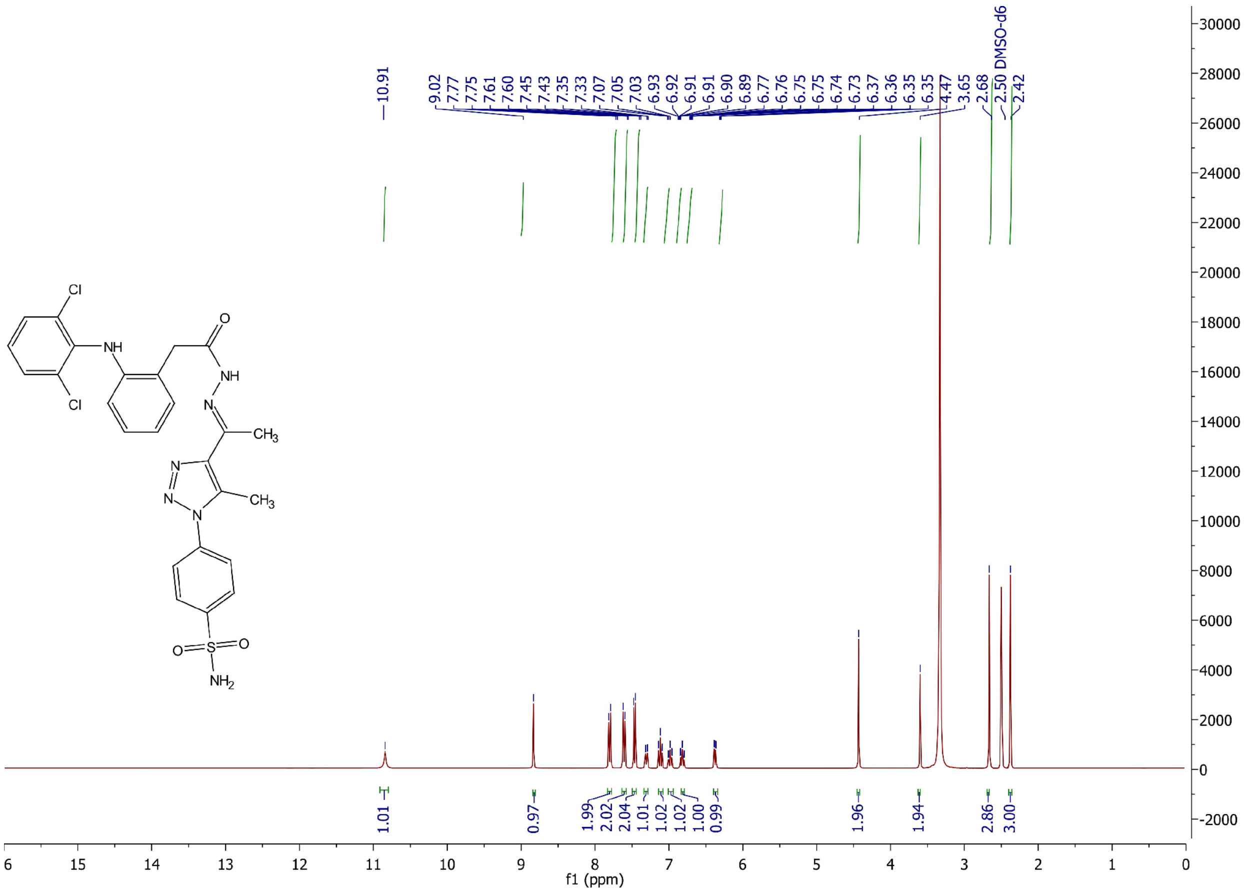

Figure S8. ^{1}H-NMR spectrum of compound 6d (DMSO-d_6)

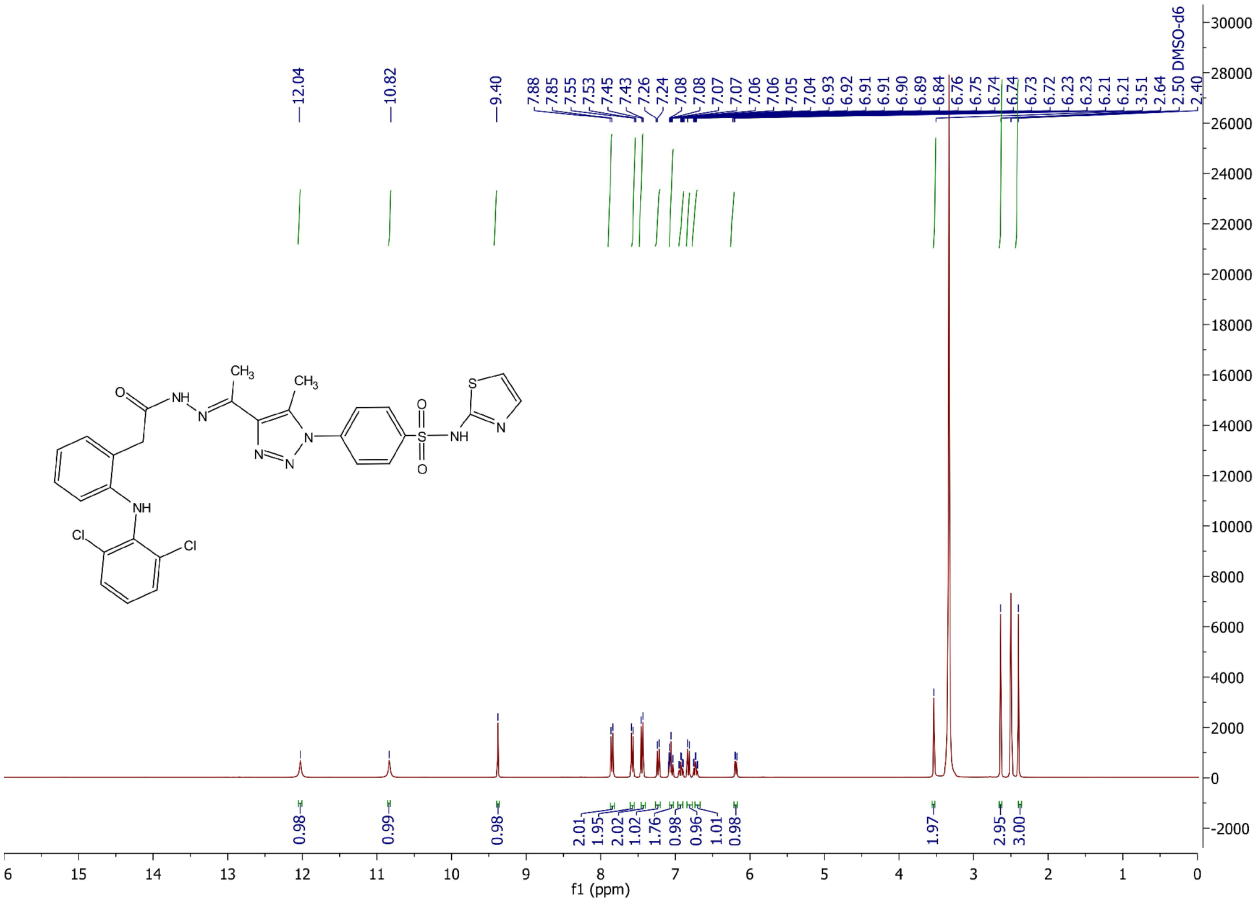

Figure S9. ^{1}H-NMR spectrum of compound 6e (DMSO-d_6)

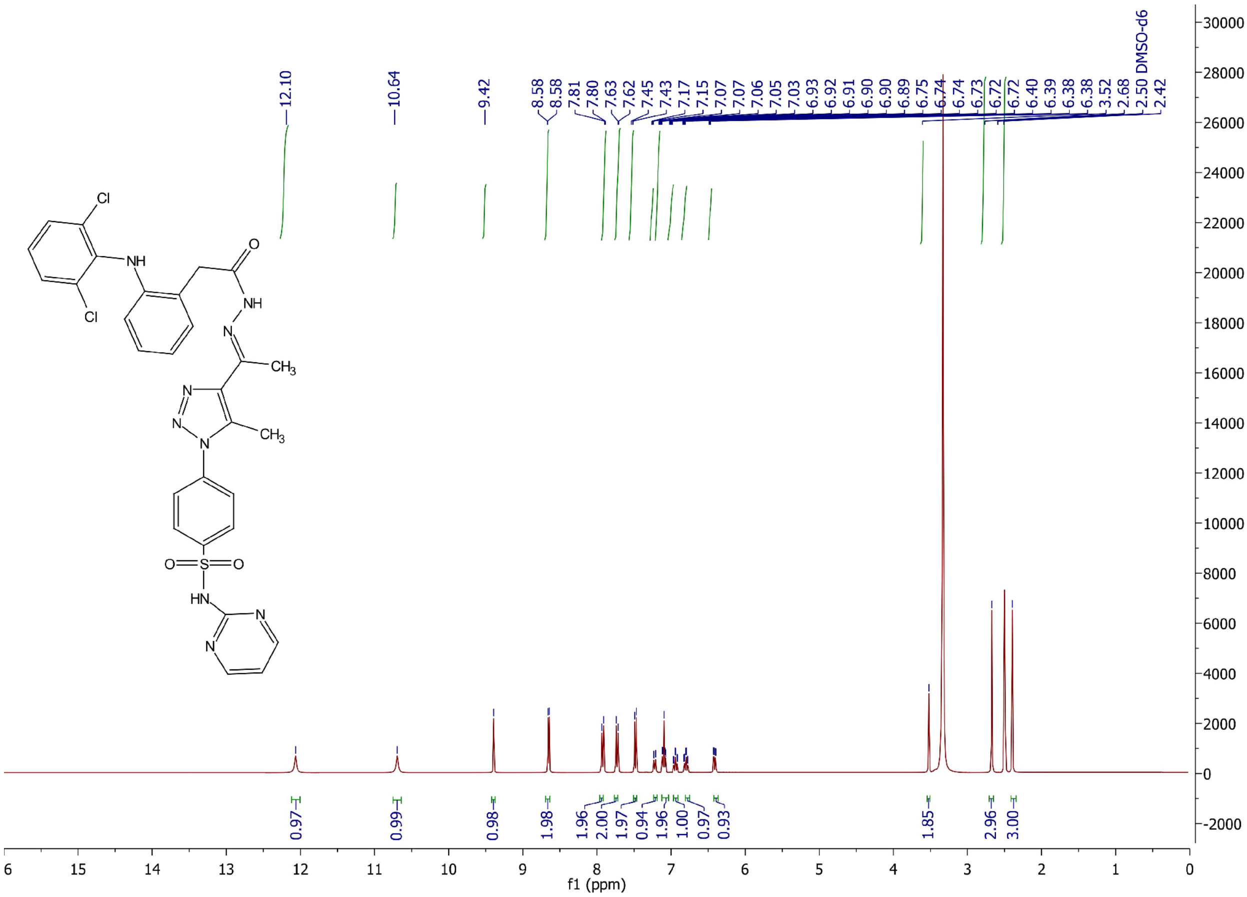

Figure S10. ^{1}H-NMR spectrum of compound 6f (DMSO-d_6)

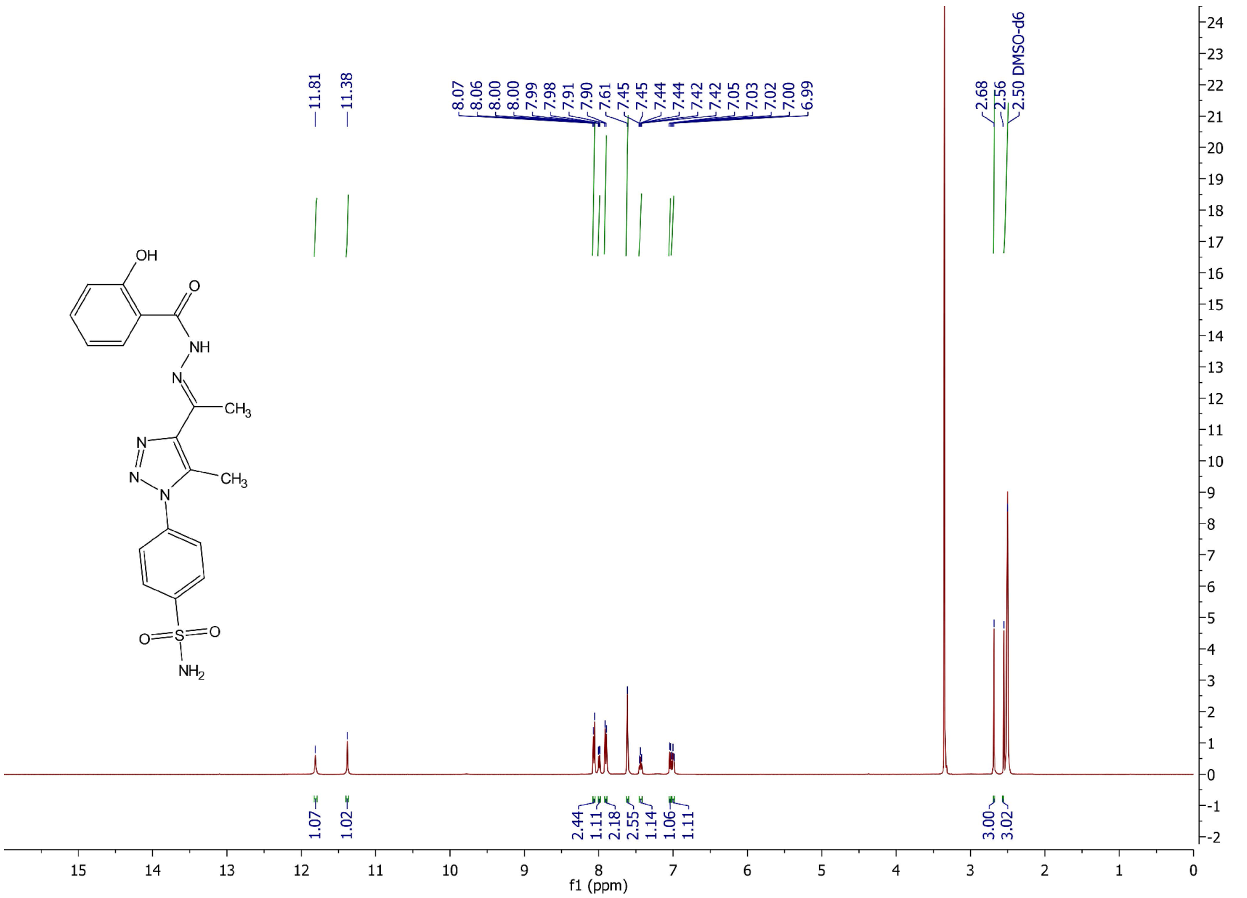

Figure S11. ^{1}H-NMR spectrum of compound 6g (DMSO-d_6)

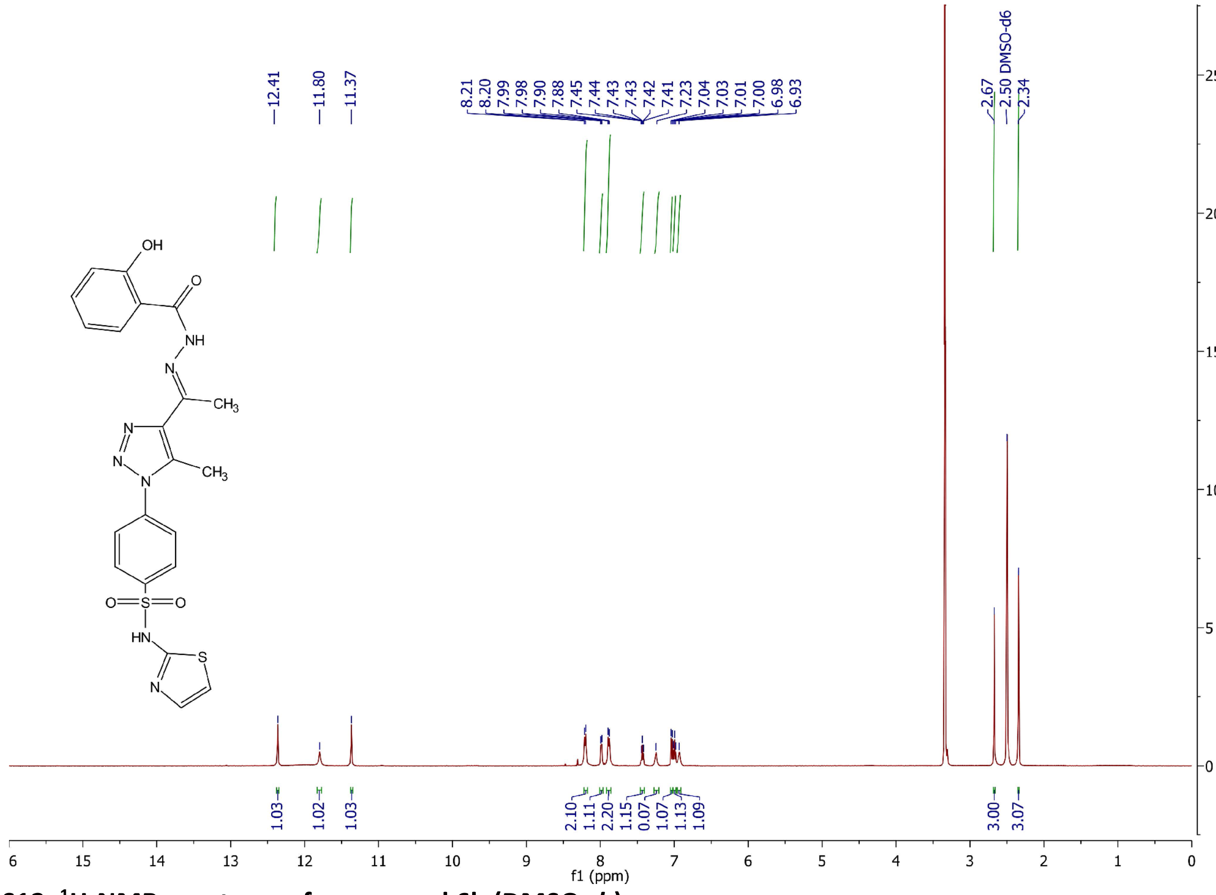

Figure S12. ^{1}H-NMR spectrum of compound 6h (DMSO-d_6)

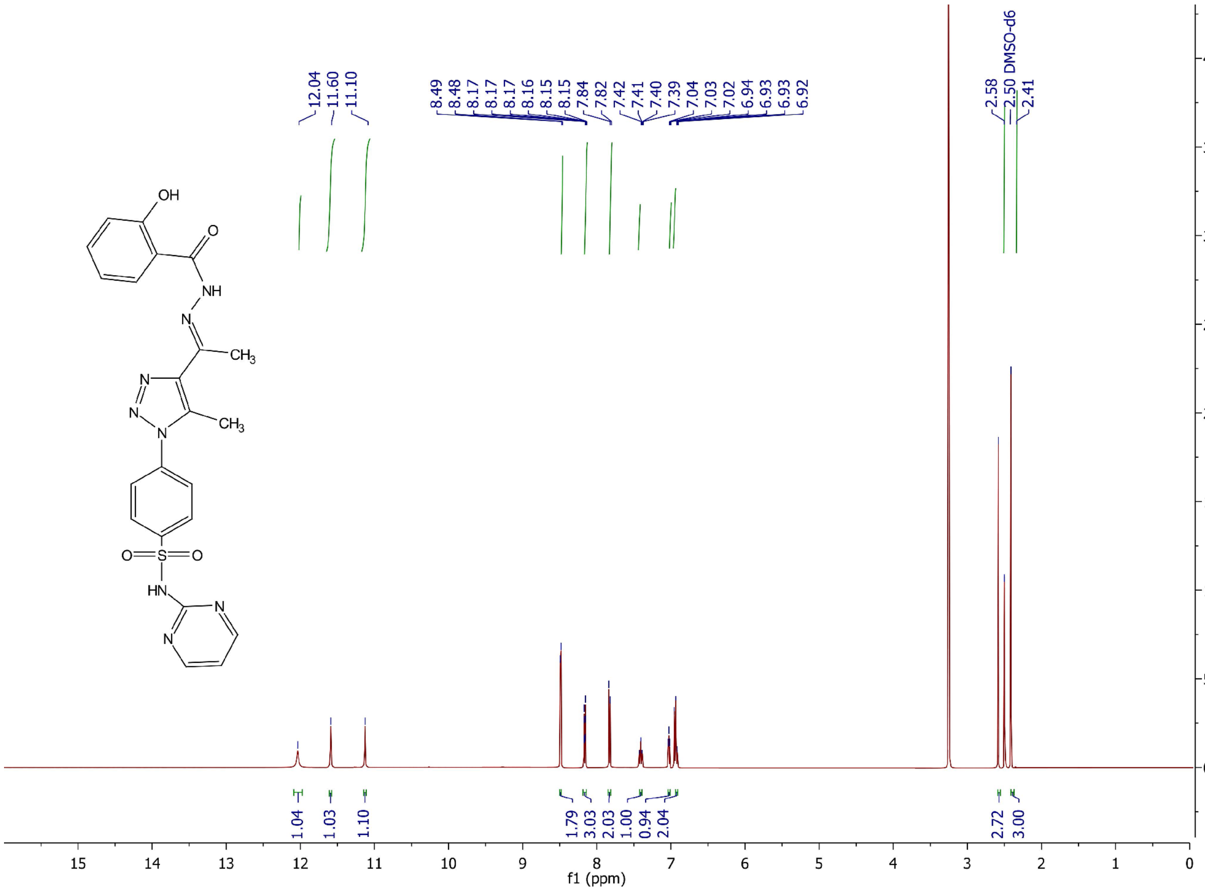

Figure S13. ^{1}H-NMR spectrum of compound 6i (DMSO-*d*$_6$)

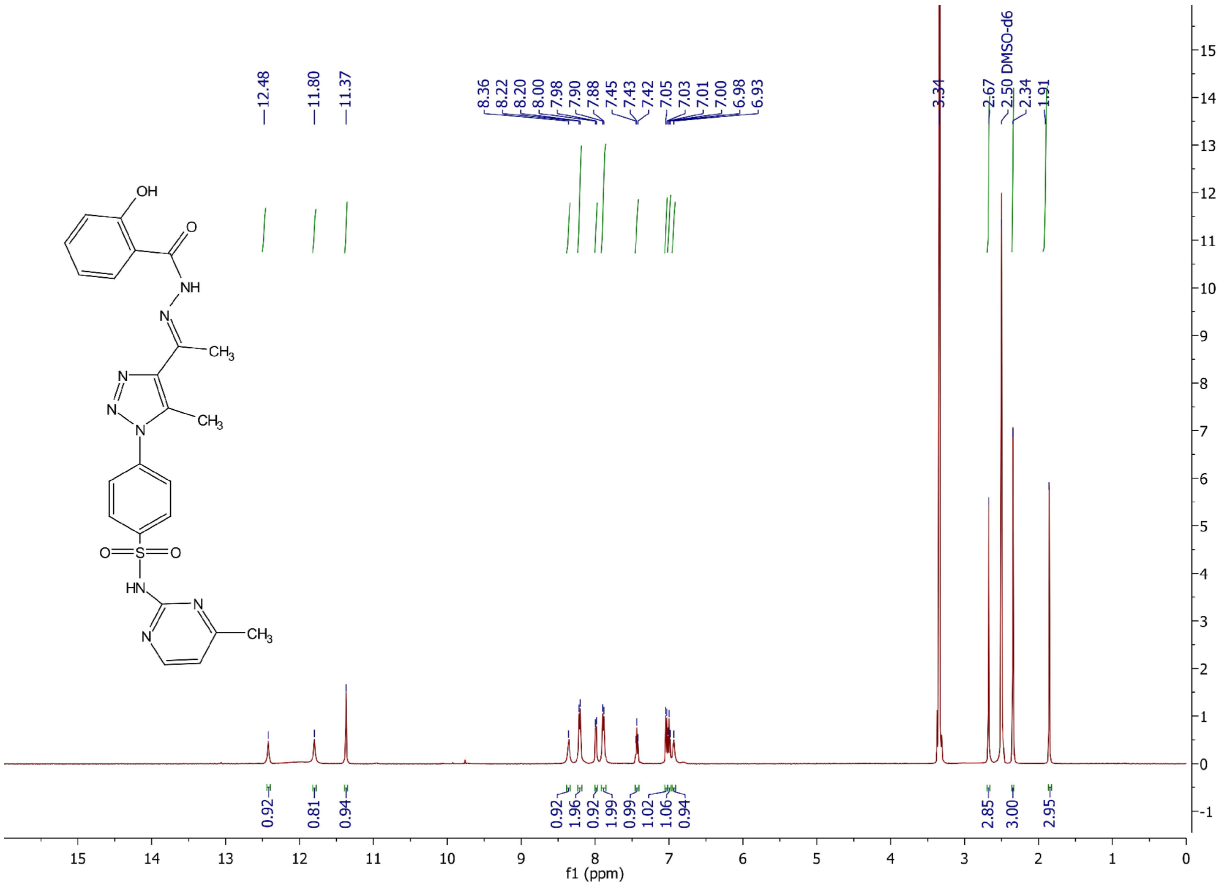

Figure S14. ^{1}H-NMR spectrum of compound 6j (DMSO-*d*$_6$)

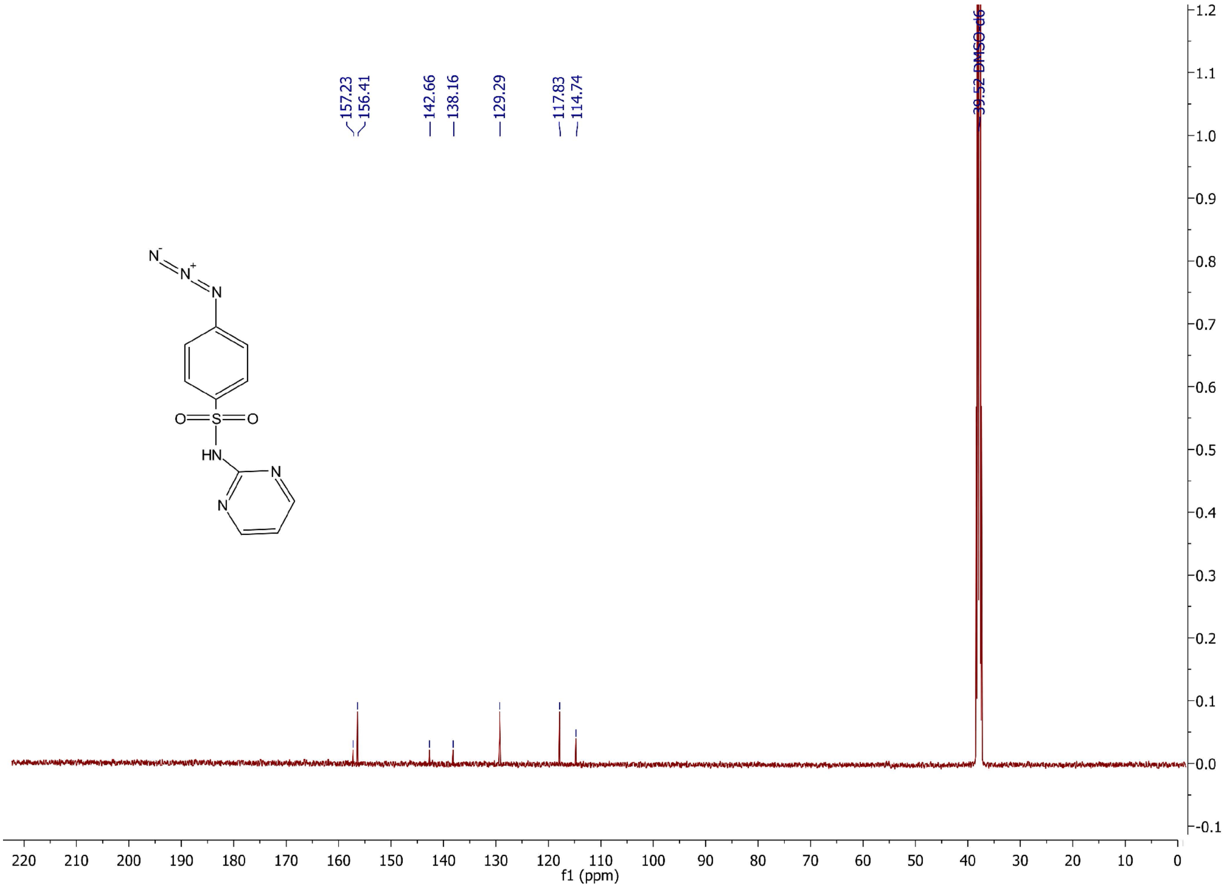

Figure S15. **^{13}C-NMR spectrum of compound 1c (DMSO-*d$_6$*)**

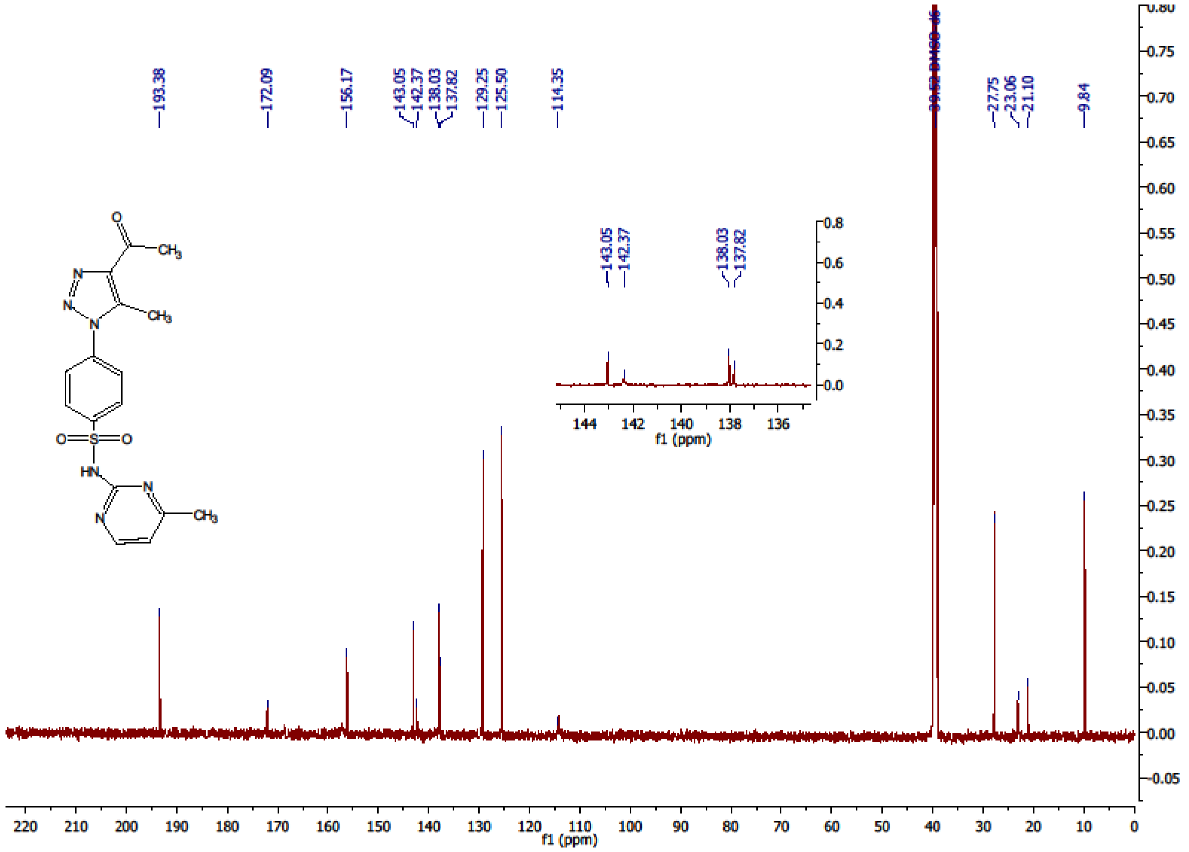

Figure S16. **^{13}C-NMR spectrum of compound 4d (DMSO-*d$_6$*)**

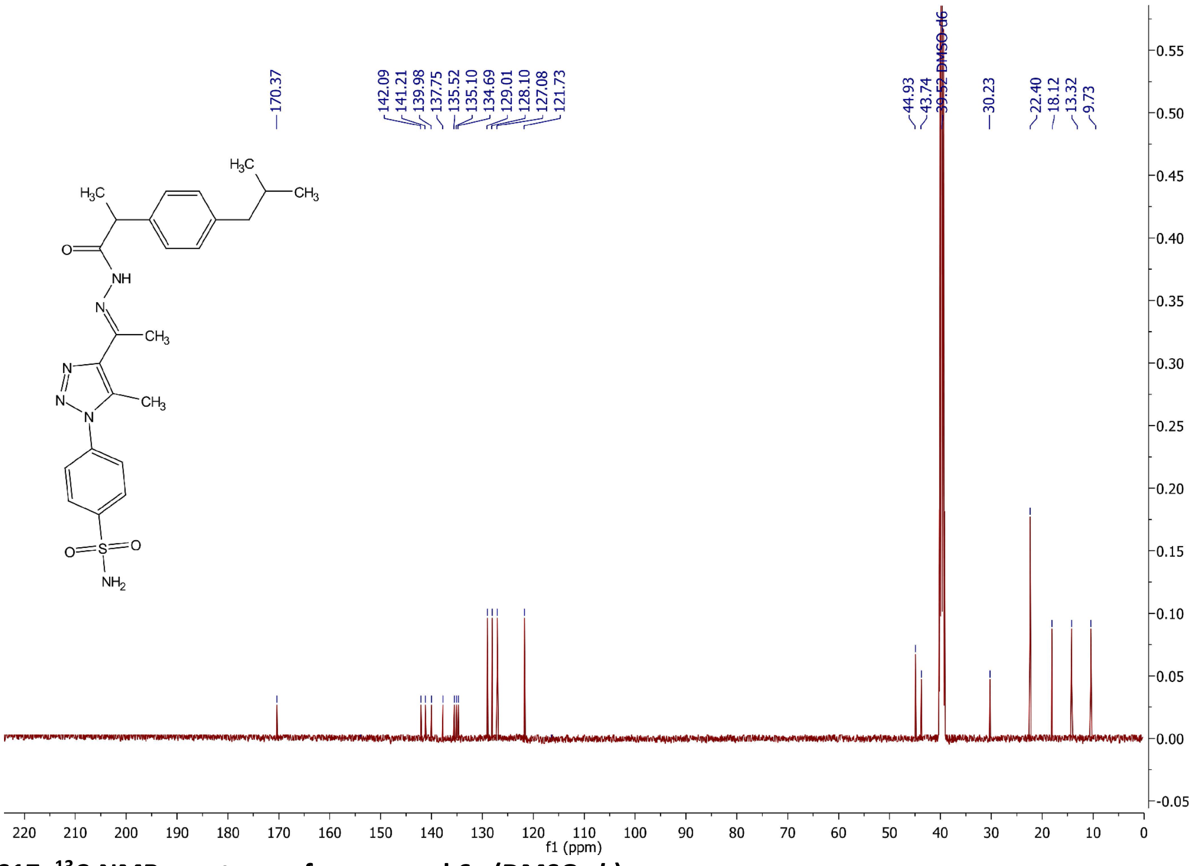

Figure S17. ^{13}C-NMR spectrum of compound 6a (DMSO-*d₆*)

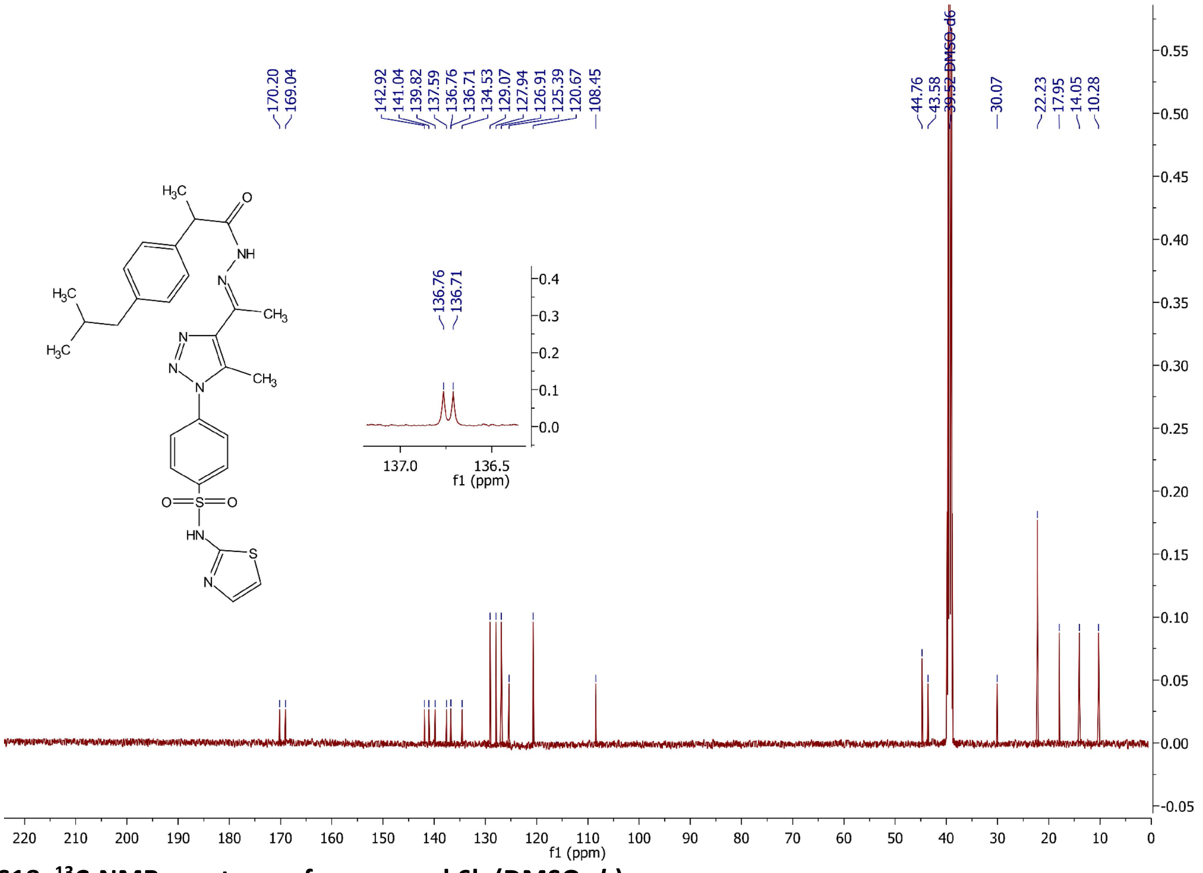

Figure S18. ^{13}C-NMR spectrum of compound 6b (DMSO-d_6)

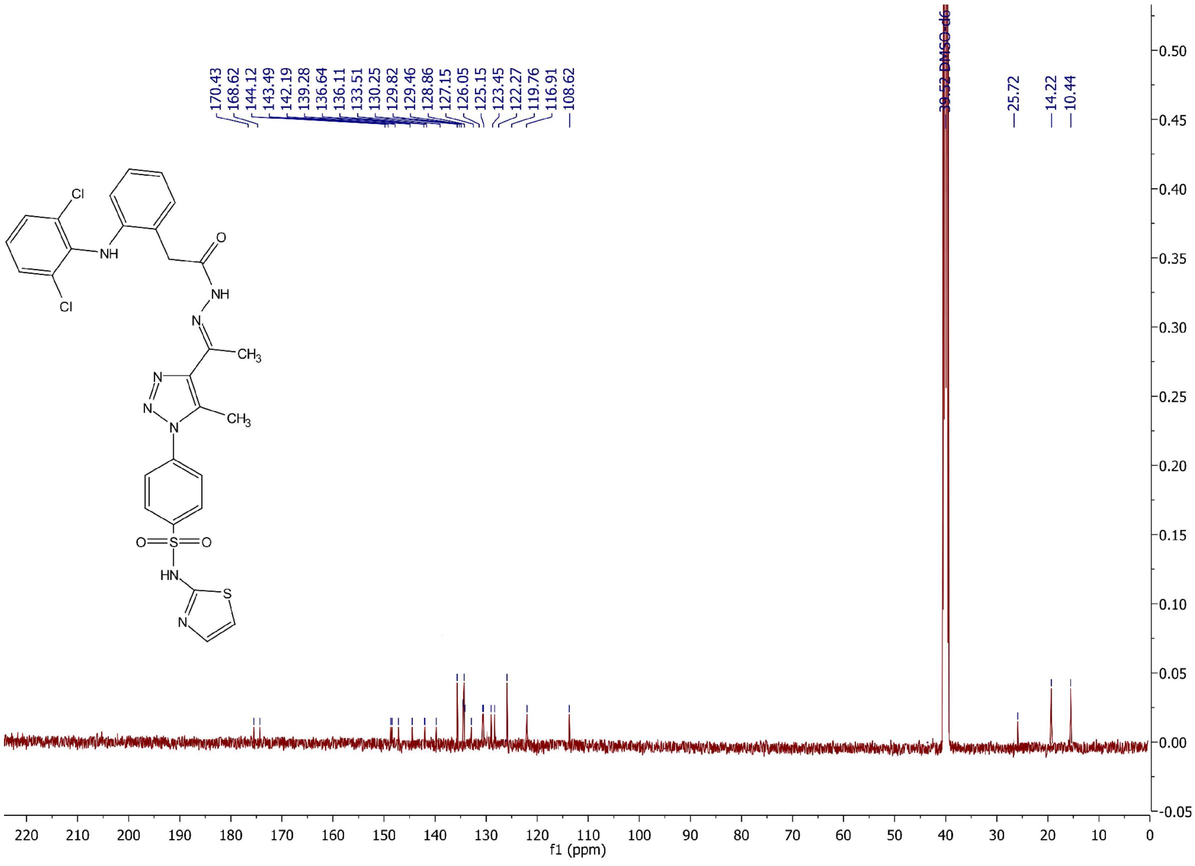

Figure S19. ^{13}C-NMR spectrum of compound 6e (DMSO-*d$_6$*)

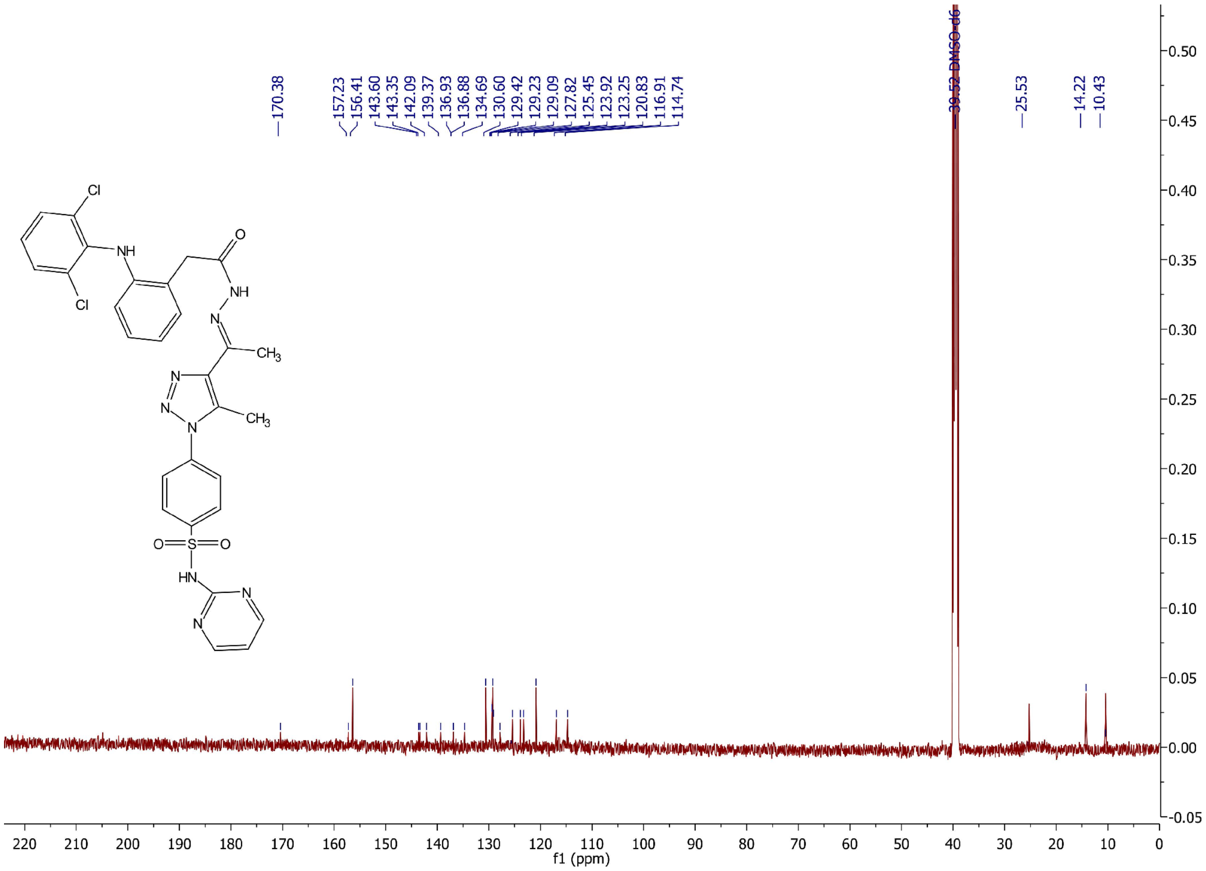

Figure S20. ^{13}C-NMR spectrum of compound 6f (DMSO-*d$_6$*)

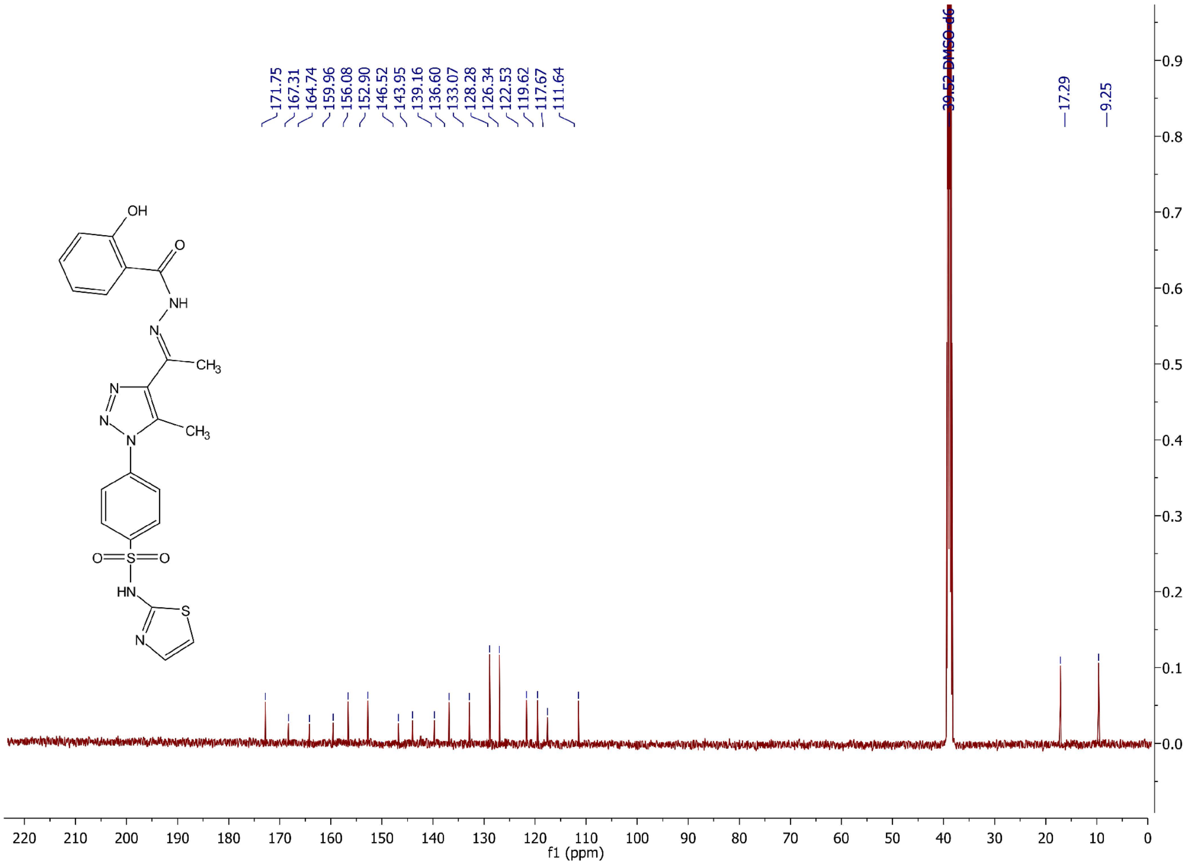

Figure S21. ^{13}C-NMR spectrum of compound 6h (DMSO-*d*$_6$)

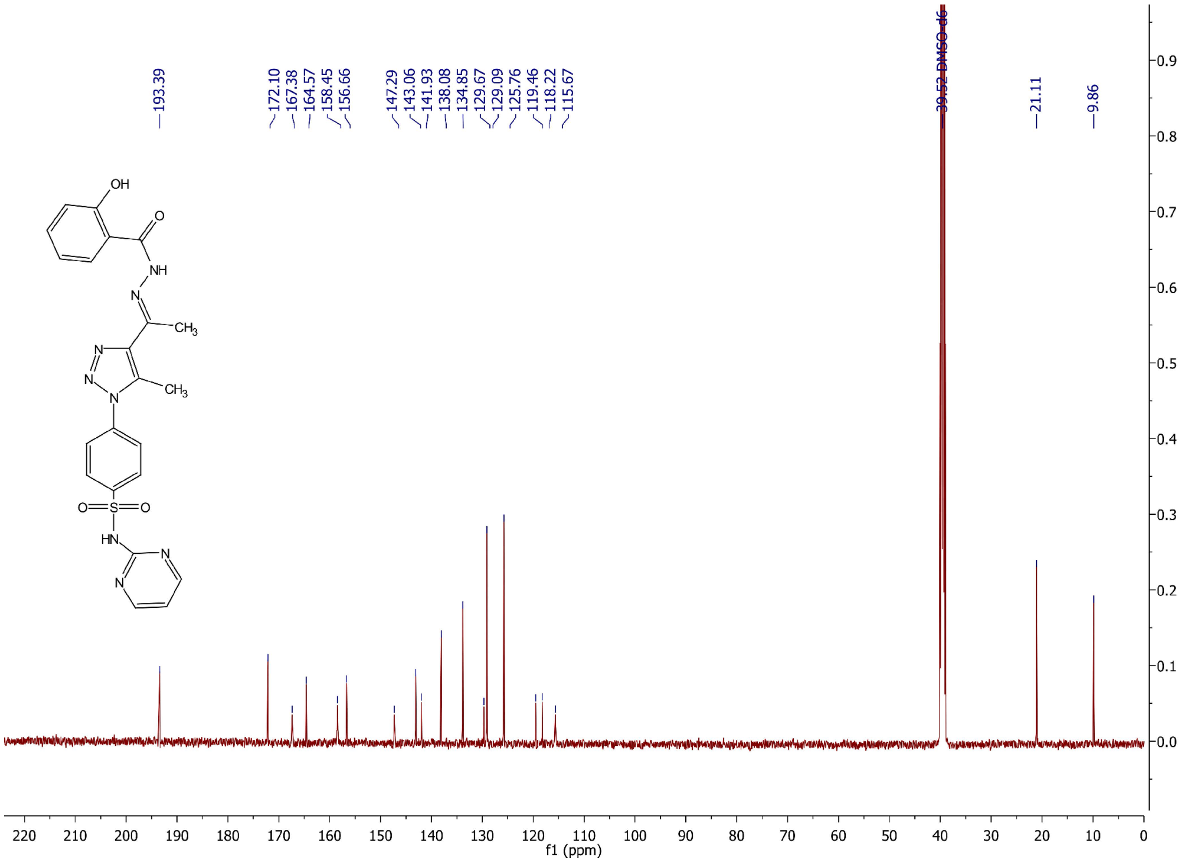

Figure S22. ^{13}C-NMR spectrum of compound 6i (DMSO-*d*$_6$)

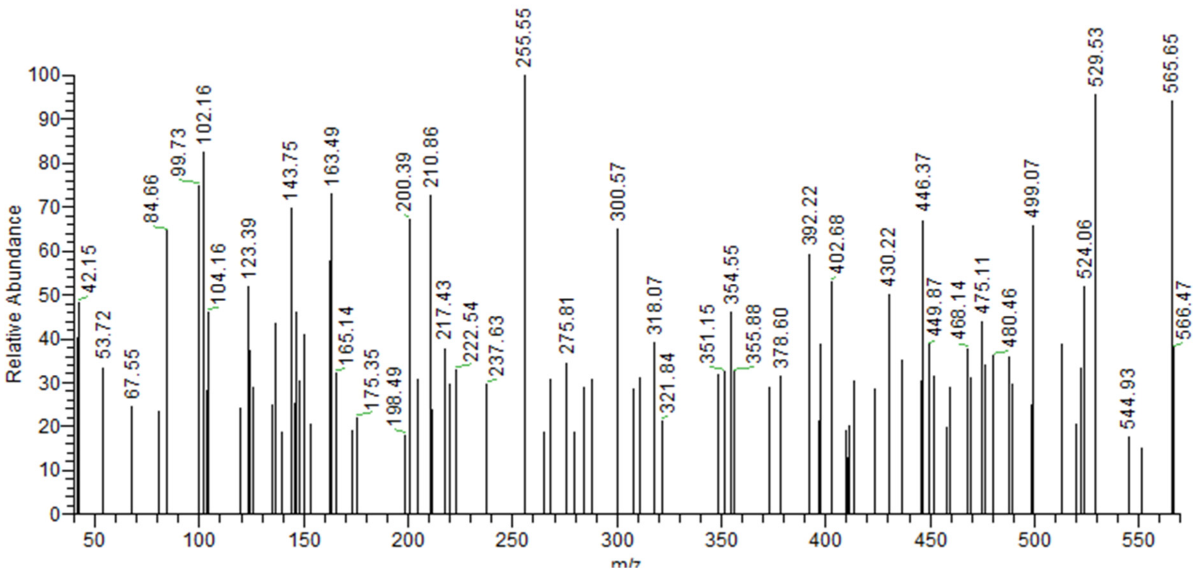

Figure S23. EI-MS spectrum of compound 6b

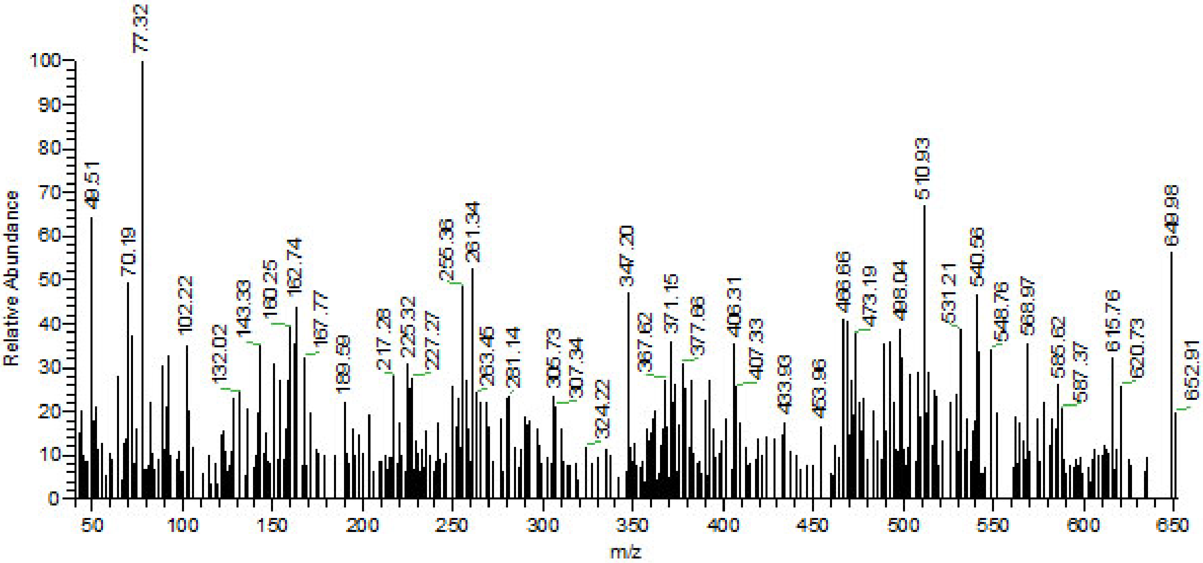

Figure S24. EI-MS spectrum of compound 6f

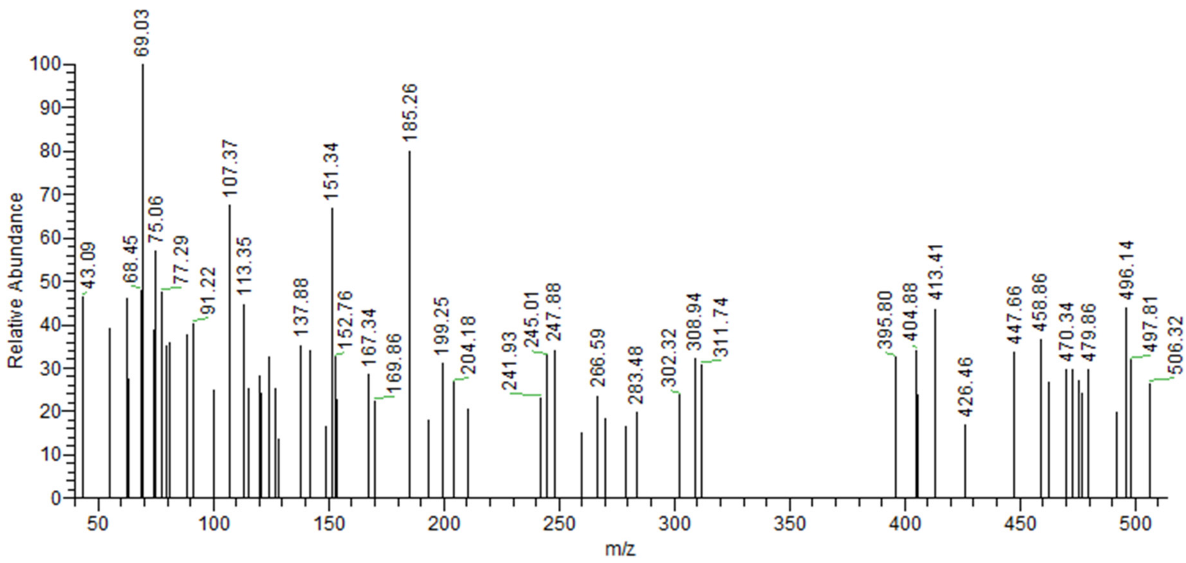

Figure S25. EI-MS spectrum of compound 6j

4.2. Biological screening

4.2.1. *In vitro* human COX-1 and human COX-2 enzymatic inhibitory activities

In vitro human COX-1 and human COX-2 enzymatic inhibitory activity of the tested compounds as well as the reference drugs was carried out according to the previously mentioned procedures [1][2]depending on Cayman colorimetric COX (ovine) inhibitor screening assay kit (Catalog No. 560131) supplied by Cayman chemicals, Ann Arbor, MI, USA. All reagents, test solutions and experimental procedures were performed in correspondence to both the manufacturer's instructions and previously reported methods. Briefly, 1 µ mL of different concentrations of tested compound solutions were added to a series of supplied reaction buffer solutions (960 µL, 0.1 M Tris-HCl, pH 8.0 containing 5 mM EDTA and 2 mM phenol) with either COX-1 or COX-2 (10 µL) enzyme in the presence of heme (10 µL). 10 µL of Arachidonic Acid (100 µM) solution were added to the mixtures of the enzyme and tested compounds, After incubating for 5 min at 25 ˚C followed by stopping COX reaction by adding of 50 µL of 1 M HCl. Moreover, after adding 100 µL of saturated stannous chloride solution, the absorbance was measured spectrophotometrically using plate reader. measuring the concentration performing 50 % enzyme inhibition (IC_{50}) was carried out by determining the percentage of human COX-1 or human COX-2 enzymatic inhibition of five different concentrations of each compound and then concentration-inhibition response curve were plotted to obtain IC_{50} values(three determinations).

4.2.2. Carrageenan-induced paw edema in mice

Male Swiss mice weighing 20 – 25 g were housed in a 12 h light/dark cycle under standard conditions as regarded to humidity (50 ± 10 %) and temperature (24 – 26 ˚C) with free access to food and water one week before performing the experiments. Groups of five mice each were subjected to induction of edema in the right-hand paw by injecting subcutaneously 0.1 mL of 1 % w/v carrageenan (Sigma-Aldrich, USA) suspended in saline. Celecoxib and diclofenac were used as positive controls which as well as the investigated compounds were injected intraperitoneally at 10 µmol/kg body weight one hour earlier than injecting carrageenan. The control group was injected with 0.5 % sodium carboxy methyl cellulose solution only. A Vernier caliper (LETICA Scientific Instruments, Barcelona, Spain) was the tool employed for measuring thickness of paw edema in mm at 0 time and along 2, 4, 6 and 8 h intervals and comparing the thickness to that of the negative control group[3][4] (Approved by HU-

IACUC)[5]. The percentage of edema inhibition (antiinflammatory activity; AI %) of each tested group were calculated as following:

AI % = VC – VD X100/VC

Where VC is the difference in paw volume in the control group and VD is the difference in paw volume in groups treated with drugs. Data were introduced as the mean ±SEM. One-way ANOVA test followed by Tukey's Karmer post hoc test were performed to determine the significance in difference between all tested groups (multiple comparison) and between the control group and groups treated with the tested compounds.

4.2.3. Determination of ED_{50}

Dose-response curves of the compounds **6b, 6e** and **6j** were plotted after testing the compounds for inhibition of the paw edema in mice after 8 h of carrageenan injection at doses of 5, 10, 20, 30 and 40 μmol/kg body weight in order to determine their ED_{50} (median effective dose) values[6] (Approved by HU-IACUC)[5].

4.2.4. Estimation of rat serum prostaglandin E2 (PGE2)

After 8 h carrageenan injection, 5 heparinized blood samples were collected from rats and subjected to plasma separation by centrifugation, frozen and stored at 20˚C until use. The quantitative determination of PGE2 in biological fluids was carried out according to competitive immunoassay procedure using EIA PGE2 kit (Aldrich, Steinheim, Germany). The kits as well as blood samples were incubated at room temperature, and then PGE2 in the blood sample was allowed to competitively bind to a monoclonal antibody to PGE2 in kit. Excess reagents were removed followed by adding the substrate was added and incubated for short time. When a yellow color generate the optical density was measured using a microplate reader DYNATech, MR 5000 (Dynatech Industries Inc., McLean, VA) at 450 nm, and expressed in pg/ml[7] (Approved by HU-IACUC)[5].
The percentage of rat serum prostaglandin E2 inhibition was calculated as following:
Cc – Cd X100/Cc
Where Cc is the concentration of rat serum PGE2 in the control group after receiving carrageenan and Cd is the concentration of rat serum PGE2 in the groups treated with carrageenan and drugs.

4.2.5. Ulcerogenic effects

The ulcerogenic effect of the tested compounds was evaluated taking celecoxib and diclofenac as reference standards. Male albino rats weighing 100 – 120 g were fasted 12 h before preceding the experiment and grouped into six groups of five rats per each. Vehicle saline only was given to the control group, whereas the test groups were dosed orally with the test compounds as well as celecoxib and Diclofenac in two equal doses of 30 μmol/kg body weight, at 4 h interval. After 6 h from the last dose, scarifying animals was performed through diethyl ether inhalation and their stomach were isolated. An opening along the greater curvature was performed followed by washing with cold saline and subjected to microscopical examination for any signs of hemorrhage, hyperemia, gastric ulcers or erosions by a 3-magnifying lens. Severity of stomach lesions was determined using an arbitrary scale that calculates the ulcer index[8] (Approved by HU-IACUC)[5].

4.3. Molecular docking studies

Molecular Operating Environment (MOE 2016.0802) software, Chemical Computing Group, Montreal, Canada was utilized in proceeding Molecular docking studies. The crystal structure of COX-2 complexed with celecoxib (PDB code 3LN1) was retrieved from RCSB Protein Date Bank website. Preparation of the compounds was performed by adding hydrogen addition and partial charges in addition to energy minimization using MMFF94x Force Field with root mean square (RMSD) gradient of 0.01 kcal/mol and RMS (Root Mean Square) distance of 0.1 Å. Moreover, COX-2 enzyme was prepared by removing water molecules, repeated chains as well as any surfactants. MOE QuickPrep protocol, 3D protonation and calculation of partial charges were carried out in order to optimize structural issues. The default procedure was applied with MOE Docking protocol parameters to select the best poses with highest binding score values of the docked compounds. Firstly, docking reliability was evaluated by extracting the co-crystallized ligand celecoxib from its active site followed by re-docking into COX-2 active site, and then molecular docking for selected compounds was performed, utilizing triangle matcher as placement method, London dG as the main scoring function and affinity dG scoring function as rigid receptor method. Furthermore, the obtained docking poses were estimated and interactions with the active site were considered. Finally, best fitting poses into the active site expressing the highest scores with the best interactions with the enzyme were chosen[9].

4.4. In silico prediction of the physicochemical properties, drug likeness score, pharmacokinetics, toxicity profile and ligand efficiency metrics

In the present study, Molinspiration chemoinformatic server was utilized for prediction of the physicochemical properties whereas, Osiris property explorer for calculating drug likeness score and toxicological effects and pharmacokinetics by Pre-ADMET calculator.

References:

[1] E.M. Gedawy, A.E. Kassab, A.M. El Kerdawy, Design, synthesis and biological evaluation of novel pyrazole sulfonamide derivatives as dual COX-2/5-LOX inhibitors, Eur. J. Med. Chem. 189 (2020) 112066. https://doi.org/https://doi.org/10.1016/j.ejmech.2020.112066.

[2] N.H. El-Dershaby, S.A. El-Hawash, S.E. Kassab, H.G. Dabees, A.E. Abdel Moneim, I.A. Abdel Wahab, M.M. Abd-Alhaseeb, M.M.M. El-Miligy, Rational design of biodegradable sulphonamide candidates treating septicaemia by synergistic dual inhibition of COX-2/PGE2 axis and DHPS enzyme, J. Enzyme Inhib. Med. Chem. 37 (2022) 1737–1751. https://doi.org/10.1080/14756366.2022.2086868.

[3] M.M.M. El-Miligy, A.K. Al-Kubeisi, S.R. El-Zemity, R.A. Nassra, M.M. Abu-Serie, A.A. Hazzaa, Discovery of small molecule acting as multitarget inhibitor of colorectal cancer by simultaneous blocking of the key COX-2, 5-LOX and PIM-1 kinase enzymes, Bioorg. Chem. 115 (2021) 105171. https://doi.org/https://doi.org/10.1016/j.bioorg.2021.105171.

[4] Y. El-Dash, N.A. Khalil, E.M. Ahmed, M.S.A. Hassan, Synthesis and biological evaluation of new nicotinate derivatives as potential anti-inflammatory agents targeting COX-2 enzyme, Bioorg. Chem. 107 (2021) 104610. https://doi.org/https://doi.org/10.1016/j.bioorg.2020.104610.

[5] N.H. El-Dershaby, S.A. El-Hawash, S.E. Kassab, H.G. Daabees, M.M.M. El-Miligy, A.E.A. Moneim, Design, Synthesis and Biological Evaluation of Some Novel Sulfonamide Derivatives as Dual Antiinflammatory and Antibacterial Agents, Institutional Anim. Care Use Comm. (HU-IACUC), Fac. Sci. Helwan Univ. Egypt, Approv. No. HU2021/Z/AE0721-01. (n.d.).

[6] S.E. Kassab, M.A. Khedr, H.I. Ali, M.M. Abdalla, Discovery of new indomethacin-based analogs with potentially selective cyclooxygenase-2 inhibition and observed diminishing to

PGE2 activities., Eur. J. Med. Chem. 141 (2017) 306–321. https://doi.org/10.1016/j.ejmech.2017.09.056.

[7] Y. Shibaike, M. Gotoh, C. Ogawa, S. Nakajima, K. Yoshikawa, T. Kobayashi, K. Murakami-Murofushi, 2-Carba cyclic phosphatidic acid inhibits lipopolysaccharide-induced prostaglandin E2 production in a human macrophage cell line, Biochem. Biophys. Reports. 19 (2019) 100668. https://doi.org/https://doi.org/10.1016/j.bbrep.2019.100668.

[8] M.S. Abouzeit-Har, T. Verimer, J.P. Long, Effect of long term estrogen and lithium treatment on restraint induced gastric erosion in intact and ovariectomized rats, Pharmazie. 37 (1982) 593–595. http://europepmc.org/abstract/MED/6183681.

[9] M.M.M. El-Miligy, A.A. Hazzaa, H. El-Messmary, R.A. Nassra, S.A.M. El-Hawash, New benzothiophene derivatives as dual COX-1/2 and 5-LOX inhibitors: synthesis, biological evaluation and docking study, Future Med. Chem. 9 (2017) 443–468. https://doi.org/10.4155/fmc-2016-0230.

HELWAN
UNIVERSITY

IACUC

APPROVAL CERTIFICATE

THIS IS TO CERTIFY THAT

Institutional Animal Care and Use Committee (HU-IACUC) Faculty of Science, Helwan University,

Approval Number: HU2021/Z/AEN0721-01

Research Title: Design,synthesis and biological evaluation of some novel sulfonamide derivatives as dual antiinflammatory and antibacterial agents

The Main and Co-Supervisor Names: Nada Hassan El-dershaby, Soad A. El-Hawash, Shaymaa E. Kassab Hoda G. Daabees, Mostafa M.M. El-Miligy, and Ahmed E. Abdel Moneim **have followed the rules of the ethical committee.**

Head of HU-IACUC
Prof. Dr. Rami Kassab

Head of Zoology and Entomology Department
Prof. Dr. Ahmed Esmat Abdel Moneim
